# *Brassica napus* Genome Possesses Extraordinary High Number of *CAMTA* Genes and *CAMTA3* Contributes to PAMP Triggered Immunity and Resistance to *Sclerotinia sclerotiorum*

**DOI:** 10.3389/fpls.2016.00581

**Published:** 2016-05-04

**Authors:** Hafizur Rahman, You-Ping Xu, Xuan-Rui Zhang, Xin-Zhong Cai

**Affiliations:** ^1^Institute of Biotechnology, College of Agriculture and Biotechnology, Zhejiang UniversityHangzhou, China; ^2^Center of Analysis and Measurement, Zhejiang UniversityHangzhou, China

**Keywords:** *Brassica napus*, *CAMTA*, disease resistance, PAMP triggered immunity, *Sclerotinia sclerotiorum*

## Abstract

Calmodulin-binding transcription activators *(CAMTAs)* play important roles in various plant biological processes including disease resistance and abiotic stress tolerance. Oilseed rape (*Brassica napus* L.) is one of the most important oil-producing crops worldwide. To date, compositon of *CAMTAs* in genomes of *Brassica* species and role of *CAMTAs* in resistance to the devastating necrotrophic fungal pathogen *Sclerotinia sclerotiorum* are still unknown. In this study, 18 *CAMTA* genes were identified in oilseed rape genome through bioinformatics analyses, which were inherited from the nine copies each in its progenitors *Brassica rapa* and *Brassica oleracea* and represented the highest number of *CAMTAs* in a given plant species identified so far. Gene structure, protein domain organization and phylogentic analyses showed that the oilseed rape *CAMTAs* were structurally similar and clustered into three major groups as other plant *CAMTAs*, but had expanded subgroups *CAMTA3* and *CAMTA4* genes uniquely in rosids species occurring before formation of oilseed rape. A large number of stress response-related *cis*-elements existed in the 1.5 kb promoter regions of the *BnCAMTA* genes. *BnCAMTA* genes were expressed differentially in various organs and in response to treatments with plant hormones and the toxin oxalic acid (OA) secreted by *S. sclerotiorum* as well as the pathogen inoculation. Remarkably, the expression of *BnCAMTA3A1* and *BnCAMTA3C1* was drastically induced in early phase of *S. sclerotiorum* infection, indicating their potential role in the interactions between oilseed rape and *S. sclerotiorum*. Furthermore, inoculation analyses using Arabidopsis *camta* mutants demonstrated that *Atcamta3* mutant plants exhibited significantly smaller disease lesions than wild-type and other *Atcamta* mutant plants. In addition, compared with wild-type plants, *Atcamta3* plants accumulated obviously more hydrogen peroxide in response to the PAMP chitin and exhibited much higher expression of the CGCG-box-containing genes *BAK1* and *JIN1*, which are essential to the PAMP triggered immunity (PTI) and/or plant resistance to pathogens including *S. sclerotiorum*. Our results revealed that *CAMTA3* negatively regulated PTI probably by directly targeting *BAK1* and it also negatively regulated plant defense through suppressing JA signaling pathway probably via directly targeting *JIN1*.

## Introduction

Calcium is a ubiquitous second messenger used by plants to regulate a variety of biological processes in response to a wide range of environmental and developmental stimuli (Galon et al., 2010; Reddy et al., 2011). In response to these stimuli, Ca^2+^ signals are decoded and transmitted by several types of Ca^2+^ sensor proteins including calmodulins (CaMs), calcineurin B-like proteins (CBLs), and calcium-dependent protein kinases (CDPKs/CPKs; Kudla et al., 2010; Du et al., 2011). CaM can bind to certain transcription factors such as calmodulin-binding transcription activators *(CAMTAs)*.

*CAMTAs*, also referred to as signal-responsive (SR) proteins, are thought to exist in all multicellular organisms (Bouché et al., 2002; Rahman et al., 2016). Taking advantage of the rapid developing of plant genome sequencing, *CAMTA* family has been identified at genome-wide level in over 40 plant species (Bouché et al., 2002; Choi et al., 2005; Koo et al., 2009; Yang et al., 2012; Shangguan et al., 2014; Wang et al., 2015; Yang et al., 2015; Yue et al., 2015; Rahman et al., 2016). Nevertheless, composition of *CAMTAs* in many economically important crop species such as *Brassica* species is still unknown.

*CAMTAs* contain multiple functional domains including a CG-1 DNA-binding domain, an ankyrin (ANK) repeat domain, an IQ (Isoleucine glutamine) domain, and a CaM binding (CaMB) domain that are located in turn from the N terminus to the C terminus (Bouché et al., 2002; Choi et al., 2005; Finkler et al., 2007; Rahman et al., 2016). Most of *CAMTAs* also possess a TIG (Transcription-associated immuno globulin-like) domain (Rahman et al., 2016). *CAMTAs* specifically recognize and bind to (A/C/G)CGCG(T/C/G) or (A/C)CGTGT *cis*-elements in the promoter region of target genes, thereby regulate their expression (Yang and Poovaiah, 2002; Choi et al., 2005; Du et al., 2009). The biological functions of *CAMTAs* are being revealed but mainly in Arabidopsis, rice and tomato. The functions of *CAMTAs* were dependent on their interaction with Ca^2+^/CaM (Choi et al., 2005; Du et al., 2009). Arabidopsis *CAMTA3* negatively regulates accumulation of salicylic acid and host plant resistance to both bacterial (Du et al., 2009) and fungal pathogens (Galon et al., 2008; Nie et al., 2012) as well as nonhost resistance to bacterial pathogen *Xanthomonas oryzae* pv. *oryzae*, probably via tuning *CBP60G, EDS1*, and *NDR1*-mediated defense signaling and reactive oxygen species (ROS) accumulation (Rahman et al., 2016). AtCAMTA3 signaling is modulated by ubiquitination process during regulation of plant immunity (Zhang et al., 2014). Similarly, a rice *CAMTA* OsCBT-1 negatively regulates rice resistance to blast fungal pathogen and leaf blight bacterial pathogen (Koo et al., 2009). Besides, AtCAMTA3 also plays important roles in plant defense against insect herbivore, glucose metabolism and ethylene-induced senescence in Arabidopsis (Laluk et al., 2012; Qiu et al., 2012). Arabidopsis *CAMTA1, CAMTA2*, and *CAMTA3* contribute to low temperature and freezing tolerance by activation of *CBF* (C-repeat/DRE binding factor) transcription factors (Doherty et al., 2009; Kim et al., 2013). Tomato *CAMTAs* are differentially expressed during fruit development and ripening processes and in responsive to biotic and abiotic stimuli (Yang et al., 2012, 2013). Silencing of *SlSR1* and *SlSR3L* enhances resistance to bacterial and fungal pathogens while silencing of SlSR1L leads to decreased drought stress tolerance (Li et al., 2014). Collectively, these reports clearly demonstrate that *CAMTAs*, especially *CAMTA3*, are important regulators of plant resistance to biotrophic pathogens. Nevertheless, their role in plant resistance to necrotrophic pathogens remains poorly understood.

Oilseed rape (*Brassica napus* L.) is one of the most important oil crops worldwide. Despite relatively extensive studies of *CAMTAs* in several model plant species, little is known about this gene family in oilseed rape and other *Brassica* species. Only one *CAMTA* sequence has been identified in oilseed rape to date (Bouché et al., 2002). In this study, taking advantage of completion of the oilseed rape genome sequence (Chalhoub et al., 2014), we systemically identified the *CAMTA* gene family in *B. napus* genome and performed comprehensive sequence analyses as well as functional analyses in disease resistance. Our results demonstrated that oilseed rape genome contained the highest number of *CAMTAs* in a given plant species identified so far. *BnCAMTA3A1* and *BnCAMTA3C1* were likely to be the functional homologs of *AtCAMTA3* functioning in disease resistance. Furthermore, using Arabidopsis *camta* mutants, we revealed that *CAMTA3* negatively regulated chitin-triggerred immunity and plant defense to the devastating necrotrophic pathogen *Sclerotinia sclerotiorum*, probably via directly targeting *BAK1* and *JIN1*.

## Materials and methods

### Identification of *CAMTA* proteins in *Brassica* species

To identify *CAMTA* protein sequences in oilseed rape, the six Arabidopsis *CAMTAs* were used as query to search by BLASTP program against *B. napus* genome databases deposited in NCBI (http://www.ncbi.nlm.nih.gov/) and the GNEOSCOPE (http://www.genoscope.cns.fr/spip/). All retrieved non-redundant sequences were collected, and subjected to conserved domain analysis using the Pfam (http://pfam.sanger.ac.uk/) and NCBI-CDD (http://www.ncbi.nlm.nih.gov/cdd) databases. These sequences were compared with Arabidopsis and tomato CAMTA proteins using ClustalW2 program (http://www.ebi.ac.uk/Tools/msa/clustalw2/) with default settings and were viewed by GeneDoc. Those containing a CG-1 domain, an ANK repeat domain and a CaMB domain were recognized as CAMTA proteins. CAMTAs in oilseed rape were named in accordance with their phylogenetic relationship to six Arabidopsis CAMTAs. Identification of CAMTAs in *B. rapa* and *B. oleracea*, two progenitor species of *B. napus*, was performed similarly.

### Gene structure, protein domain, and phylogenetic analyses of *BnCAMTA* genes

The gene structure was analyzed online by the Gene Structure Display Server (GSDS, http://gsds.cbi.pku.edu.cn/index.php; Guo et al., 2007). A schematic diagram of protein domain structures with functional motifs was constructed using Domain Illustrator software (http://dog.biocuckoo.org/; Ren et al., 2009). The sequence logos of CaMB domain were generated using the Geneious software (v6.1.6) package (http://www.geneious.com/). Multiple sequence alignments of the full-length CAMTA proteins from representative plant species were conducted using ClustalW. The phylogenetic tree was constructed using MEGA 5.0 (Tamura et al., 2011) with maximum likelihood (ML) method and a bootstrap test was performed with 1000 replicates.

### Prediction of *cis*-acting elements in the *BnCAMTA* genes

To investigate *cis*-elements in the promoter sequences of the *BnCAMTA* genes, 1.5 kb sequences upstream of the initiation codon (ATG) were collected and subjected to stress response-related *cis*-acting element online prediction analysis with Signal Scan search program in the PLACE database (http://www.dna.affrc.go.jp/PLACE/signalscan.html) and the PlantCARE database (http://bioinformatics.psb.ugent.be/webtools/plantcare/html/).

### Plant material and hormone treatments

Oilseed rape plants were grown in growth room at 22–23°C with a 16/8 h day/night photoperiod. Arabidopsis plants of Col-0 and six *CAMTA* mutants (*Atcamta1-6*) were grown in a growth chamber at 20–21°C under a 15/9 h day/night photoperiod. For *BnCAMTA* gene expression analyses, leaves of 4-week-old plants were sprayed with hormones SA (1 mM) and JA (200 μM) as well as a chemical OA (1 mM), the toxin secreted by the pathogen *S. sclerotiorum*, or 0.01% ethonal (the solvent for the above chemicals) as a control, and collected at 0, 4, 12, and 24 h after treatment. In addition, various organs of oilseed rape plants including root, stem, cotyledone, and true leaves were also sampled for gene expression analysis. All samples were immediately frozen in liquid nitrogen and stored at −80°C until RNA extraction.

### *S. sclerotiorum* inoculation and plant resistance analyses

Leaves of 4 week-old *B. napus* and Arabidopsis plants were inoculated with mycelial plugs of 3 mm diameter of *S. sclerotiorum* as described (Saand et al., 2015a). The inoculated leaves were collected at 0, 6, and 12 h post inoculation for RNA extraction and gene expression analyses. The necrosis symptoms of the inoculated leaves were investigated and the size of lesions was measured. The inoculation analysis was performed three times, each in at least 6 plants for each treatment and gene backgrounds. For the statistical analysis of the lesion size data, ANOVA (analysis of variance) analysis was performed with SPSS software (Version 19.0, IBM, USA). Significant difference between the mean values of three independent experiments was determined with Duncan's multiple range test (DMRT; *p* < 0.05).

### Detection of chitin-triggered hydrogen peroxide

The hydrogen peroxide (H_2_O_2_) elicited by chitin (100 μg mL^−1^, Sigma, USA) in leaf discs of *Atcamta3* mutant and wild type Col-0 plants were measured using a Microplate Luminometer (TITERTEK BERTHOLD, Germany) following previously described protocol (Saand et al., 2015a). For each experiment, 10 leaves were collected for each genotype. All experiments were conducted three times independently. The quantitative measurement data were statistically analyzed using SPSS software and represent means ± standard error.

### RNA isolation and gene expression analyses

Total RNA was extracted with Trizol reagent (TAKARA, Japan) following the manufacturer's instructions. RNA was treated with DNase I (TAKARA, Japan) and reverse-transcribed into cDNA using the PrimeScript RT reagent kit (TAKARA, Japan). The obtained cDNAs were used for gene expression analyses with semiquantitative reverse transcription PCR (RT-PCR) and quantitative real time PCR (qRT-PCR). Semiquantitative RT-PCR was performed following the program: 94°C for 5 min, followed by 32 or 28 (for internal control gene) cycles of denaturation for 50 s at 94°C, annealing for 50 s at 55°C, extension for 20 s at 72°C, and a final extension for 10 min at 72°C. The obtained products were analyzed by electrophoresis on a 1.5% agarose gel and detected under ultraviolet light. The qRT-PCR was conducted in StepOne Real-Time PCR System (Applied Biosystems, USA) using SYBER Premix Ex Taq reagents (TaKaRa, Japan) following the program: 95°C for 30 s, 95°C for 5 s, and 60°C for 45 s for 40 cycles. To normalize the sample variance, *B. napus* β *-Tubulin* and Arabidopsis *ACTIN8* genes served as internal controls. Relative gene expression values were calculated using the 2^−ΔΔCt^ method. The primers used for gene expression analyses are listed at Table S1. For the statistical analysis of the gene expression data, ANOVA analysis was performed with SPSS software (Version 19.0, IBM, USA). Significant difference between mean values was determined with DMRT (*p* < 0.05).

## Results

### Identification of *CAMTA* genes in *B. napus* and its two progenitor species

**Figure 1:**
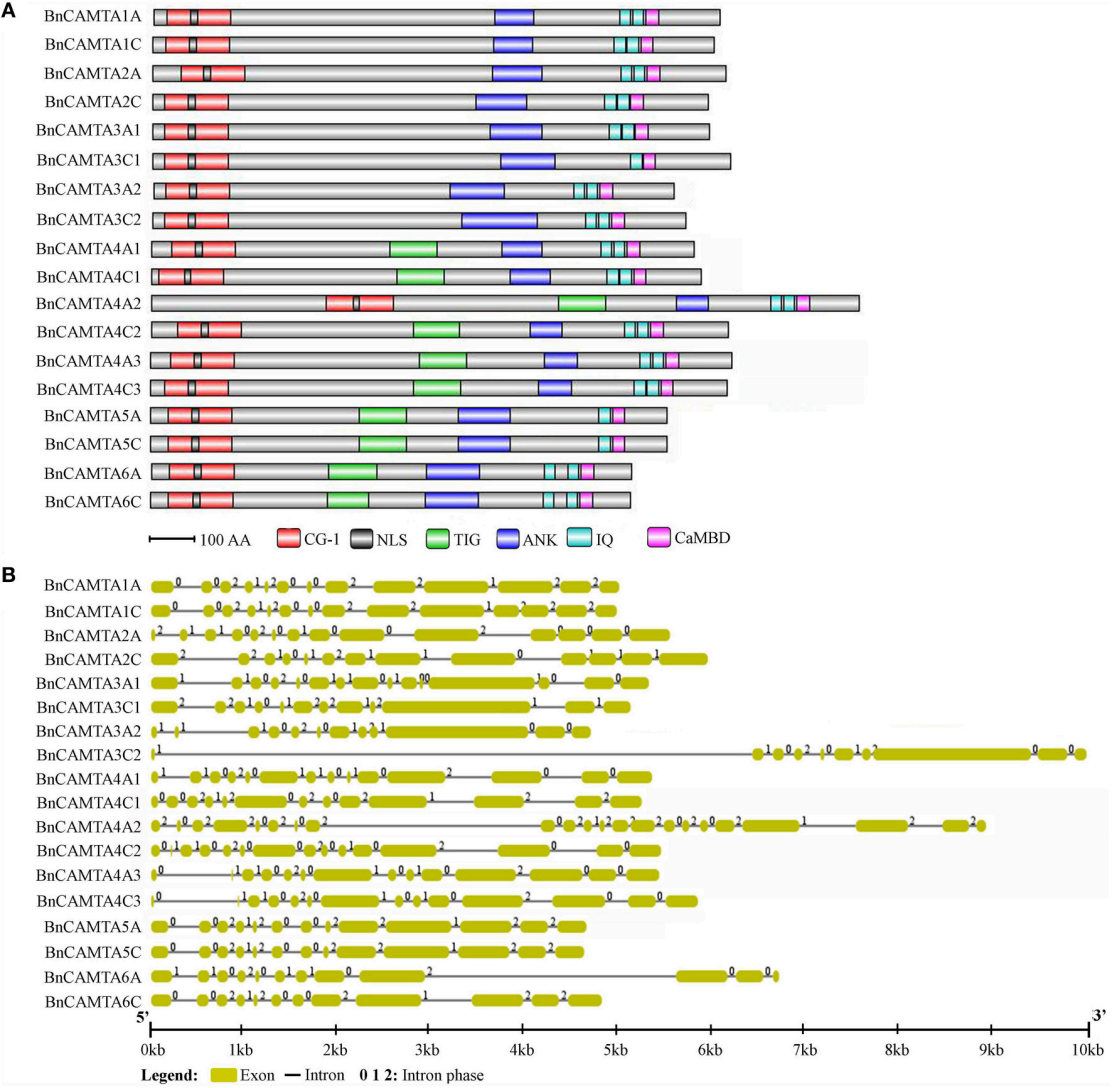
**Protein domain composition and gene structure of BnCAMTAs**. **(A)** Schematic representation of domains of BnCAMTA proteins. Analyses of conserved domains were performed using the Pfam database (http://pfam.janelia.org/). NLS motifs were searched by Motif scan (http://myhits.isb-sib.ch/cgi-bin/motif_scan). CaMBDs were analyzed using the Calmodulin Target Database (http://calcium.uhnres.utoronto.ca/ctdb/ctdb/). The domain structures of BnCAMTAs were drawn to scale using Domain Graph software (http://dog.biocuckoo.org/). Abbreviations: CG-1, CG-1 DNA-binding domain; NLS, nuclear localization signal motif; TIG, transcription-associated immuno globulin-like domain; ANK, ankyrin repeat domain; IQ, isoleucine glutamine motif; CaMBD, calmodulin-binding domain. **(B)** Exon-intron structure of *BnCAMTA* genes. The exons and introns are indicated by blue boxes gray lines, respectively. The *BnCAMTA* gene structures were drawn to scale using the Structure Display Server (GSDS, http://gsds.cbi.pku.edu.cn/).

To identify *CAMTA* genes in *B. napus*, the six Arabidopsis CAMTAs were used as query to BLASTP search in the complete genome of *B. napus*. Based on domain composition analyses for the retrieved candidate sequences, a total of 18 CAMTA sequences were identified in *B. napus* genome, representing the highest number of CAMTAs in a given plant species identified so far. They were named in accordance with their phylogenetic relationship with the six Arabidopsis CAMTAs and the location in subgenomes (A or C). The comprehensive information of *BnCAMTA* genes, including locus ID, gene location, length and intron number, predicted protein size, molecular weight, and isoelectric point (pI), is listed in Table 1. The length of the *BnCAMTA* gene sequences was 4.6–6.0 kb with three exceptions *BnCAMTA3C2* (10.0 kb), *BnCAMTA4A2* (8.9 kb), and *BnCAMTA6A* (6.7 kb), which contained significantly longer genomic sequence due to possessing an extraordinarily large intron (Table 1; Figure 1). The size of predicted BnCAMTA proteins was 919–1034 amino acids (aa) except BnCAMTAs 6A and 6C with 853 aa and BnCAMTA4A2 with 1258 aa (Table 1). BnCAMTA4A2 was larger due to carrying an extra N terminal sequence (Figure 1A). BnCAMTA proteins varied obviously in their pI value. The majority of them (12 out of 18) owned a pI of lower than 6.2, two of them (BnCAMTAs 6A and 6C) had a pI of near 7.0, while the remaining four (BnCAMTAs 4C2, 4A2, 5A, and 5C) possessed a pI of higher than 7.4 (Table 1), implying that while most of the BnCAMTA proteins are acidic, some of them are neutral or basic. Collectively, these results indicated that *B. napus* genome possesses much more *CAMTA* genes than other plant species, and their physico-chemical characteristics were generally conserved but with obvious exceptions.

**Table 1:** ***CAMTA* gene family in oilseed rape**.

**Gene name**	**Locus ID**	**Gene location**	**Gene length (bp)**	**No. of introns**	**No. of amino acid (aa)**	**Mol.Wt. (kDa)**	**pI**
Bn*CAMTA1A*	BnaA10g22560D	ChrA10: 15173521-15178533	5013	12	1007	113.5	5.88
Bn*CAMTA1C*	BnaC09g47120D	ChrC09: 46651396-46656382	4987	13	999	112.4	5.96
Bn*CAMTA*2A	BnaA09g06760D	ChrA09: 3297678-3303234	5557	13	1019	114	6.13
Bn*CAMTA*2C	BnaC09g06280D	ChrC09: 3766467-3772427	5961	12	987	110.2	5.99
Bn*CAMTA*3A1	BnaA04g12770D	ChrA04: 10768124-10773453	5330	14	990	111	5.49
Bn*CAMTA*3C1	BnaC04g34700D	ChrC04: 36202016-36207150	5135	11	1028	115	5.48
Bn*CAMTA*3A2	BnaA09g42730D	ChrA09: 29720801-29725508	4708	11	924	104.4	5.81
Bn*CAMTA*3C2	BnaC08g35210D	ChrC08: 33166518-33176534	10020	9	947	106.6	5.73
Bn*CAMTA*4A1	BnaA07g25100D	ChrA07: 18753935-18759298	5364	13	965	107.7	5.93
Bn*CAMTA*4C1	BnaC06g26850D	ChrC06: 28406024-28411276	5253	12	976	108.5	5.86
Bn*CAMTA*4A2	BnaA07g26320D	ChrA07: 19369075-19378014	8940	21	1258	139.5	7.94
Bn*CAMTA*4C2	BnaC06g28390D	ChrC06: 29594775-29600235	5461	14	1025	115.4	7.9
Bn*CAMTA*4A3	BnaA02g13050D	ChrA02: 7109254-7114694	5441	13	1034	115.3	5.42
Bn*CAMTA*4C3	BnaC02g45620D	ChrC02_random: 1363612-1369466	5855	13	1025	114.5	5.63
Bn*CAMTA*5A	BnaA08g06080D	ChrA08: 6008140-6012801	4662	12	919	104.4	7.59
Bn*CAMTA*5C	BnaC08g47670D	ChrC08_random: 2585648-2590284	4637	12	919	104.5	7.45
Bn*CAMTA*6A	BnaA05g22990D	ChrA05: 17452572-17459296	6725	11	853	97	6.92
Bn*CAMTA*6C	BnaC05g36450D	ChrC05: 35682877-35687702	4826	11	853	97	6.84

To better understand the composition of CAMTAs in the tetraploid *B. napus*, CAMTAs in its two progenitor species *B. rapa* and *B. oleracea* was also identified using similar approaches. The results showed that the two *Brassica* species exhibited similar *CAMTA* composition, both containing 9 CAMTAs (Table S1). Comparison analysis indicated that *B. napus* genome possessed exactly the total copies of CAMTAs in its two progenitor species.

### Chromosomal location of *BnCAMTA* gene family

The 18 *BnCAMTA* genes were mapped on 14 oilseed rape chromosomes (Figure S1). Among them, eight were scattered each on one chromosome (A02, C02_random, A04, C04, A05, C05, A08, and A010), while the remaining 10 were distributed in five chromosomes (C06, A07, C08, A09, and C09) with each two genes in one chromosome. BnCAMTAs 4C1 and 4C2 as well as BnCMTAs 4A1 and 4A2 were located nearly each other on Chromosomes C06 and A07, respectively, while BnCMTAs 2A and 3A2 as well as BnCAMTAs 1C and 2C were distributed distantly on the two ends of Chromosomes A09 and C09, respectively (Figure S1). In addition, BnCAMTAs 3C2 and 5C lay in Chromosome C08 although the precise position of BnCAMTA5C in this chromosome remained unclear (tentatively called ChrC08_random). This result suggested that gene duplication and recombination occurred, most obviously for *BnCAMTA4s*, and contributed to *CAMTA* gene expansion in *B. napus*.

### Conserved domain and gene structural analyses of BnCAMTAs

The CAMTA proteins consist of multiple predicted functional domains, evolutionally conserved in amino acid sequence and organization order. The domain structure analyses revealed that all the 18 BnCAMTA proteins contained a CG-1 DNA-binding domain in the N-terminal portion, an ankyrin repeat (ANK) domain in the middle, one or two IQ motifs and a calmodulin binding (CaMB) domain in the C-terminal region (Figure 1A). In addition, 10 BnCAMTAs belonging to subgroups 4, 5, and 6 contained a TIG domain, located between the N-terminal CG-1 domain and the ANK domain (Figure 1A). All BnCAMTA proteins were predicted to contain a nuclear localization signal (NLS) in the N-terminus of the protein, consistent with their role as transcription factors that function in the nucleus (Figure 1A). This result indicated that the domain composition of CAMTAs in *B. napus* is similar to those in other plant species (Rahman et al., 2016).

Further, the exon-intron structure of the *BnCAMTA* genes was analyzed. The result demonstrated that the exon-intron configuration of most *BnCAMTA* genes was highly conserved with 11-14 introns, as observed for that of *CAMTA* genes in other plant species (Rahman et al., 2016). The exceptions were *BnCAMTA3C2* and *BnCAMTA4A2* genes. Both contained an intron with an unusual large size. Additionally, *BnCAMTA3C2* had only nine introns while *BnCAMTA4A2* possessed 21 introns (Figure 1B). Whether they exhibit distinct function from the others remains further study.

### Conservation of CaMB domain of BnCAMTAs

**Figure 2:**
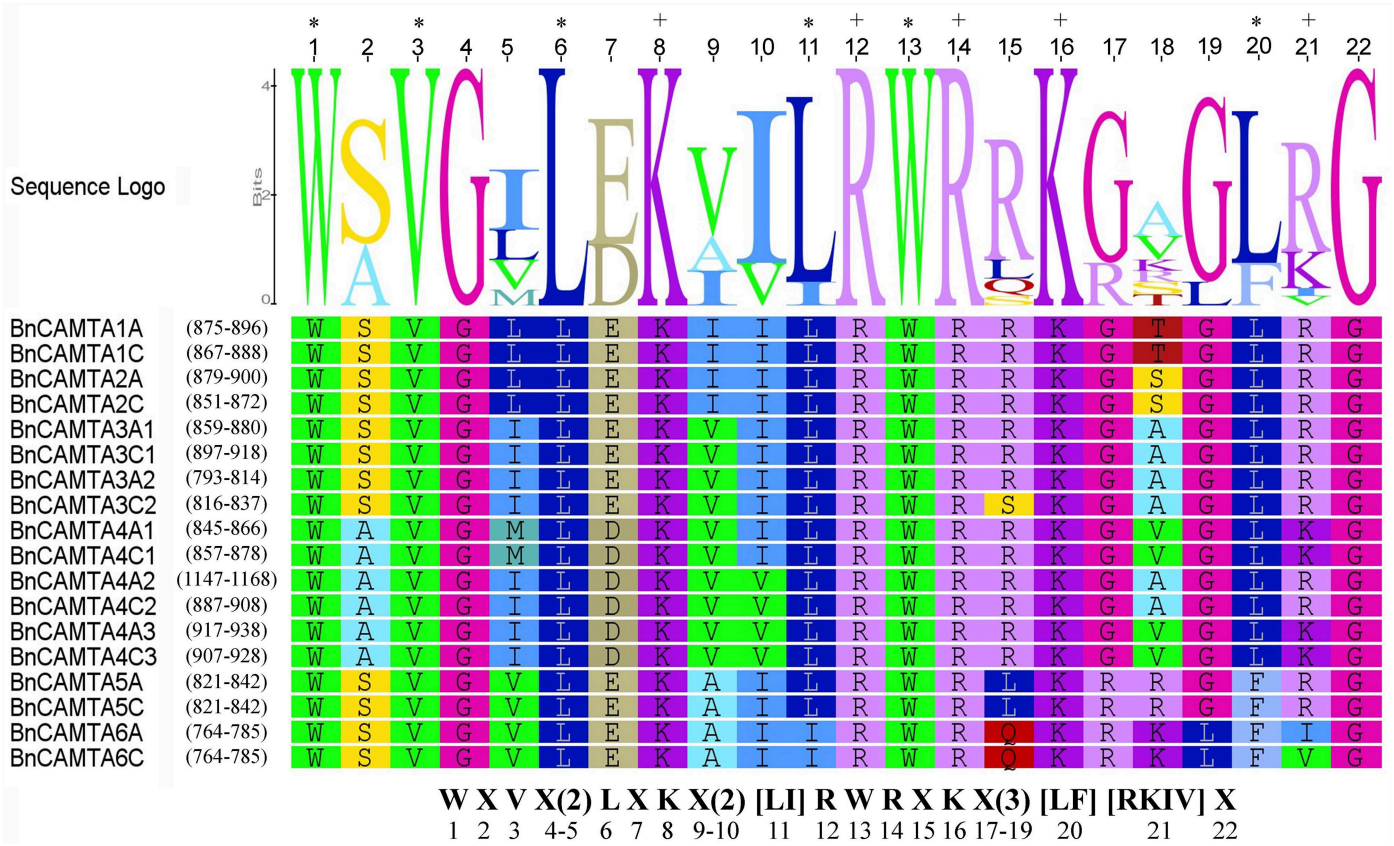
**Sequence logo of the CaMB domain of 18 BnCAMTA proteins**. The “*” and “+” indicate conserved hydrophobic and positively charged residues in the CaMB domain. The conserved motif for these functional amino acids is shown at the bottom. The sequence logo of BnCAMTAs was drawn using Geneous R6 software (v6.1.6).

CaMB domain is indispensable to CAMTAs. To understand the conservation of this domain in BnCAMTAs, the corresponding sequence regions were aligned and compared with that in well-studied Arabidopsis and tomato CAMTAs. The alignment revealed a conserved motif for functional residues as W X V X(2) L X K X(2) [LI] R W R X K X(3) [LF] [RKIV] X (Figure 2). Except for minor variation in some positions such as the 11^*th*^ and 21^*st*^ positions, the motif for BnCAMTAs generally fitted the one reported for Arabidopsis and tomato CAMTAs (W X V X(2) L X K X(2) [LF] R W R X [KR] X(3) [FL] R X). In this motif for BnCAMTAs, the 11^*th*^ hydrophobic residue was dominated by L except two sequences (BnCAMTAs 6A and 6C) as I. Similarly, the 21^*st*^ position was dominated by R, but it was K in BnCAMTAs 4A1, 4C1, 4A3, and 4C3 and I and V in BnCAMTAs 6A and 6C, respectively (Figure 2). Collectively, these data demonstrated that the motif of CaMB domain was highly conserved in CAMTA proteins of oilseed rape and other plant species.

### Phylogenetic relationship of *BnCAMTA* genes

**Figure 3:**
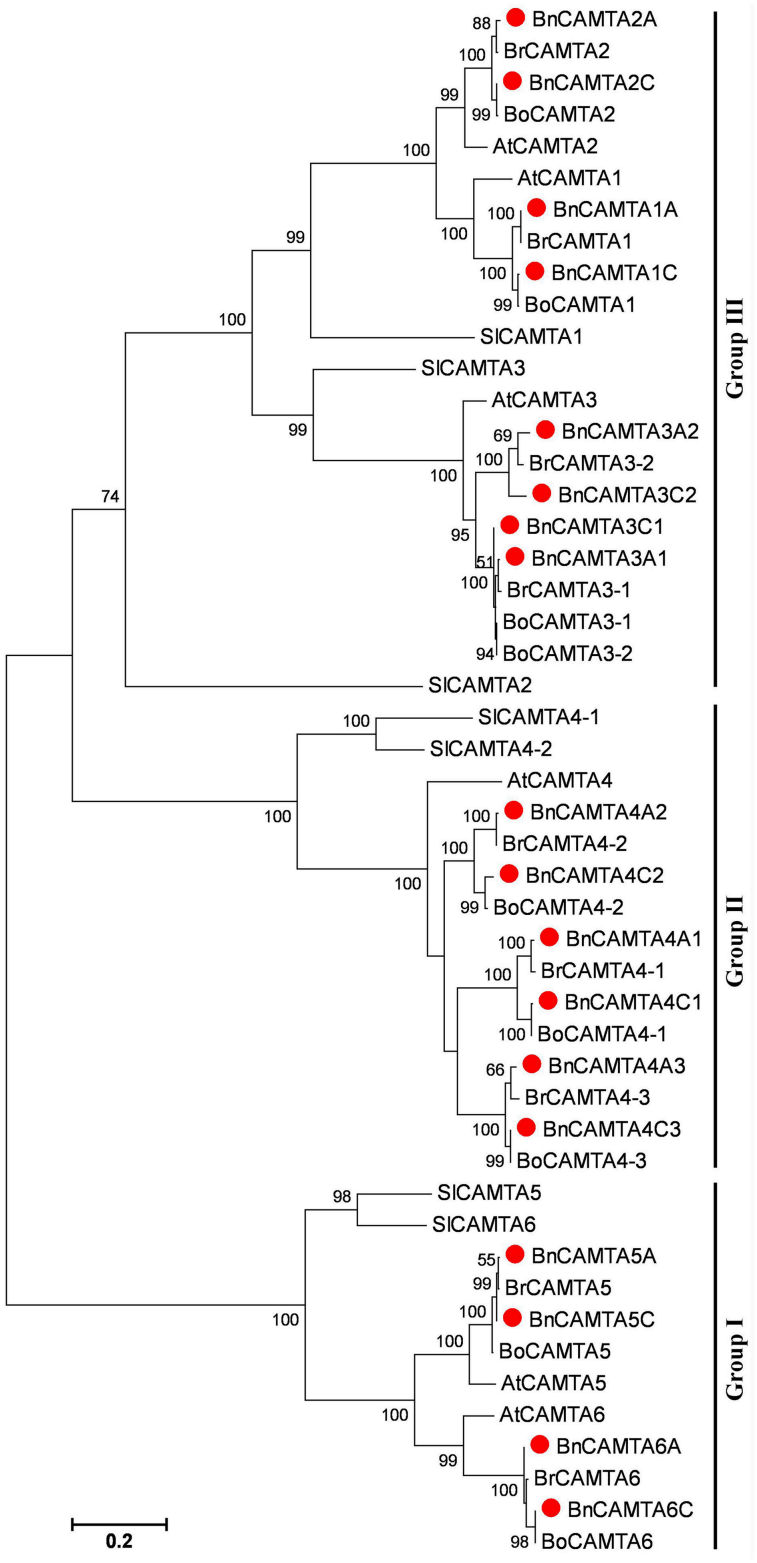
**Phylogenetic tree of oilseed rape CAMTAs along with homologs from its progenitors *B. rapa* and *B. oleracea* as well as Arabidopsis and tomato**. Bootstrap values are displayed on the branches. Oilseed rape CAMTAs are marked with a solid red circle before the protein names. The tree was generated using the MEGA5 program by maximum likelihood (ML) methods.

To gain insight into the phylogenetic relationship of *BnCAMTA* genes, a phylogenetic tree based on maximum-likelihood (ML) methods was constructed for 18 *B. napus* CAMTAs along with those from *B. rapa, B. oleracea*, Arabidopsis and tomato (Table 1, Table S2, and Figure 3). The phylogenetic analysis indicated that 18 BnCAMTAs clustered into three groups (I–III) with CAMTAs from other plant species with strong bootstrap support. All four memebers of BnCAMTA subgroups 5 and 6 constituted group I, together with CAMTA subgroups 5 and 6 from other plant species. All six members of BnCAMTA subgroup 4 gathered into group II along with CAMTA4s from other plant species, while all 8 non-TIG BnCAMTAs (all members of BnCAMTA subgroups 1, 2, and 3) formed group III, together with CAMTA subgroups 1, 2, and 3 from the other species (Figure 3). This phylogenetic tree revealed that all copies of CAMTAs in the two progenitors *B. rapa* and *B. oleracea* were well-inherited in *B. napus*. The similar clustering pattern was also obtained when the phylogenetic tree was reconstructed for the CAMTA proteins only from Arabidopsis and oilseed rape (Figure S2). It is noteworthy that different members of BnCAMTA subgroups 3 and 4 exhibited distinguishable phylogenetic distance to Arabidopsis CAMTA3 and CAMTA4. BnCAMTAs 3A1 and 3C1 were phylogenetically closer to AtCAMTA3 than BnCAMTAs 3A2 and 3C2. Similarly, BnCAMTAs 4A3 and 4C3 were phylogenetically closer to AtCAMTA4 than the other four BnCAMTA4s (Figure S2). These results indicated that *CAMTA3* and *CAMTA4* genes had been expanded in the three *Brassica* species compared with Arabidopsis although they belong to the same family (*Brassicaceae*). It is intriguing to probe whether members of the same subgroups function similarly or differentially considering that the pivotal role of AtCAMTA3 in plant defense has been unveiled.

### Prediction of *cis*-acting elements in promoters of *BnCAMTA* genes

Nine well-defined and stress response-related *cis*-acting elements (DRE/CRT, ABRE, AuxRE, SARE, G- box, W-box, CG-box, P1BS, and SURE) were scanned in 1.5 kb sequences upstream of the ATG of *BnCAMTA* genes to obtain preliminary clues on how the *BnCAMTA* genes respond expressionally to stress stimuli. The results showed that there were various stress/stimulus response-related *cis*-acting elements in the promoter of *BnCAMTA* genes (Table 2). Analyses in both PLACE and PlantCARE databases predicted that *BnCAMTA* genes widely contained ABA responsive element (ABRE) and G-box element, some carried W-box element while a few possessed auxin responsive element (AuxRE) in their promoters (Table 2). Moreover, search in PLACE database predicted that some *BnCAMTA* genes owned additional *cis*-emelmets such as dehydration and cold responsive element (DRE/CRT), auxin responsive element (AuxRE), SA-responsive element (SARE), phosphate starvation-responsive element (P1BS), and sulfur-responsive element (SURE) in their promoters (Table 2). In addition, four *BnCAMTA* genes possessed 1–3 copies of CAMTA-recognizable CG-box elements according to the prediction result in the PLACE database, suggesting that CAMTAs might regulate their own gene transcription. Interestingly, every *BnCAMTA* gene contained at least one type of stress response-related *cis*-element, but the type of *cis*-element(s) in *BnCAMTA* genes was distinguishable (Table 2). Collectively, the stress-responsive *cis*-element analyses indicated that the *BnCAMTAs* are likely to be involved in plant response to various stresses and hormone signals.

**Table 2:** **Predicted stress response-related *cis*-elements in the 1.5 kb sequence upstream of ATG of the *BnCAMTA* genes**.

**Gene Name**	**DRE/CRT**		**ABRE**		**AuxRE**		**SARE**		**G-box**		**W-box**		**CG-box**		**P1BS**		**SURE**	
	**PLACE**	**PlantCARE**	**PLACE**	**PlantCARE**	**PLACE**	**PlantCARE**	**PLACE**	**PlantCARE**	**PLACE**	**PlantCARE**	**PLACE**	**PlantCARE**	**PLACE**	**PlantCARE**	**PLACE**	**PlantCARE**	**PLACE**	**PlantCARE**
BnCAMTA1A	1						1				1				4		2	
BnCAMTA1C	4		1	1(6)	2		1		1	1(8)	2	2(6)	3		2			
BnCAMTA2A			1	4(6)					2	2(8)								
BnCAMTA2C	2		1	3(6)			3		3	3(6)	1		1					
BnCAMTA3A1				1(6)					1	1(6)					2			
BnCAMTA3C1				1(6)					1	1(6)	1	1(6)						
BnCAMTA3A2						1(7)					1				2		5	
BnCAMTA3C2						1(7)					2	1(6)	1				5	
BnCAMTA4A1									1	1(6)					2		1	
BnCAMTA4C1	2		1	1(6)	1				1	1(8)	1	1(6)					4	
BnCAMTA4A2	1		1	1(6)	1		2		1	1(6)	1						1	
BnCAMTA4C2			2	2(6)	1		1		2	2(6)								
BnCAMTA4A3			1	1(6)			1		1	1(6)	1		3					
BnCAMTA4C3			2	2(6)	3	2(6)	2				2						2	
BnCAMTA5A	1		2	4(6)	1		1		4	3(8)	1	2(6)					1	
BnCAMTA5C																	6	
BnCAMTA6A			1	1(7)							1	1(6)					1	
BnCAMTA6C	1		1	1(6)	1	1(5)			1	1(6)	1	1(6)			2			

*The Matrix score (10 at maximum) for prediction in PlantCARE database is indicated in brackets. Abbreviations for cis-elements: DRE/CRT, dehydration and cold responsive element; ABRE, ABA-responsive element; AuxRE, auxin responsive element; SARE, SA-responsive promoter element; G-box, environmental signal response element; W-box, WRKY binding site; P1BS, phosphate starvation-responsive element; SURE, sulfur-responsive element; CG-box, CAMTA binding site*.

### Constitutive expression of *BnCAMTA* genes in various tissues of *B. napus*

**Figure 4:**
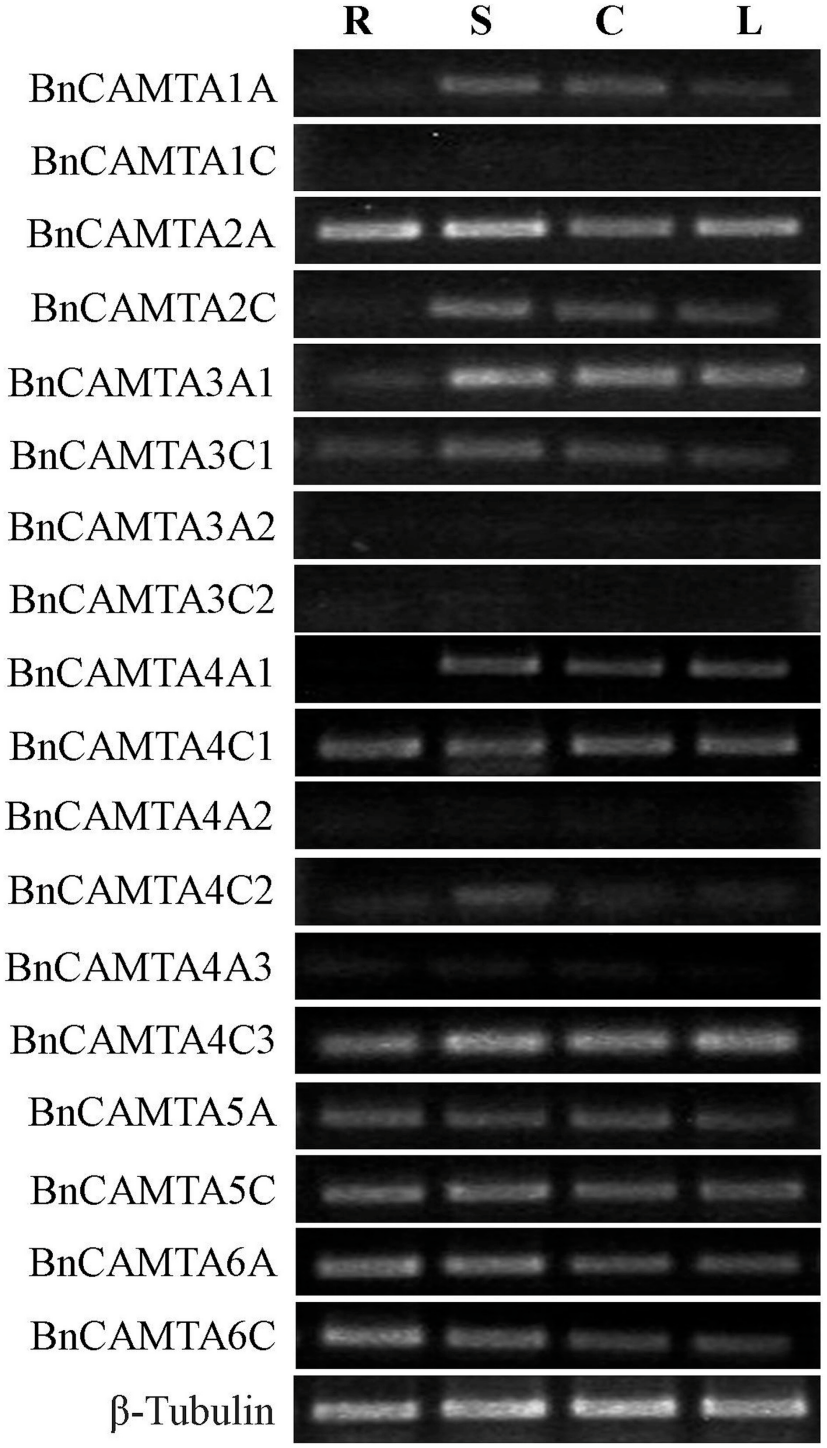
**Constitutive expression patterns of *BnCAMTA* genes in various tissues**. Expression patterns of the *BnCAMTA* genes in root (R), stem (S), cotyledon (C) and true leaf (L) were analyzed by semiquantitative RT-PCR. The oilseed rape β -Tubulin gene served as a loading control gene. The profile of electrophoresis on a 1.5% agarose gel of the products obtained from 32 cycles (28 cycles for control) of PCR was shown.

To obtain a clue for the possible functions of the *BnCAMTA* genes, their expression profiles in different tissues or organs, including cotyledons of 1-week-old seedlings as well as roots, stems, and leaves of 4-week-old plants, were analyzed by semiquantitative RT-PCR. The results showed that different *BnCAMTA* genes exhibited distinct expression patterns. Seven out of 18 *BnCAMTA* genes (*BnCAMTAs* 2A, 4C1, 4C3, 5A, 5C, 6A, and 6C) were expressed highly in all investigated organs. Five genes (*BnCAMTAs* 1A, 2C, 3A1, 3C1, and 4A1) were expressed highly in stem, cotyledon and leaves but only weakly or even not in root. *BnCAMTA4C2* gene was expressed highly in stem but weakly in all other organs, while the remaining five *BnCAMTA* genes (*BnCAMTAs* 1C, 3A2, 3C2, 4A2, and 4A3) were only very weakly expressed in all types of organs (Figure 4), since their transcripts were detected only in the second round of RT-PCR using products of the first round PCR as template (Figure S3). Collectively, these expression data provided evidence to support that *BnCAMTA* genes play distinct roles in plant development.

### Expression of *BnCAMTA* genes in response to hormone and chemical treatments

**Figure 5:**
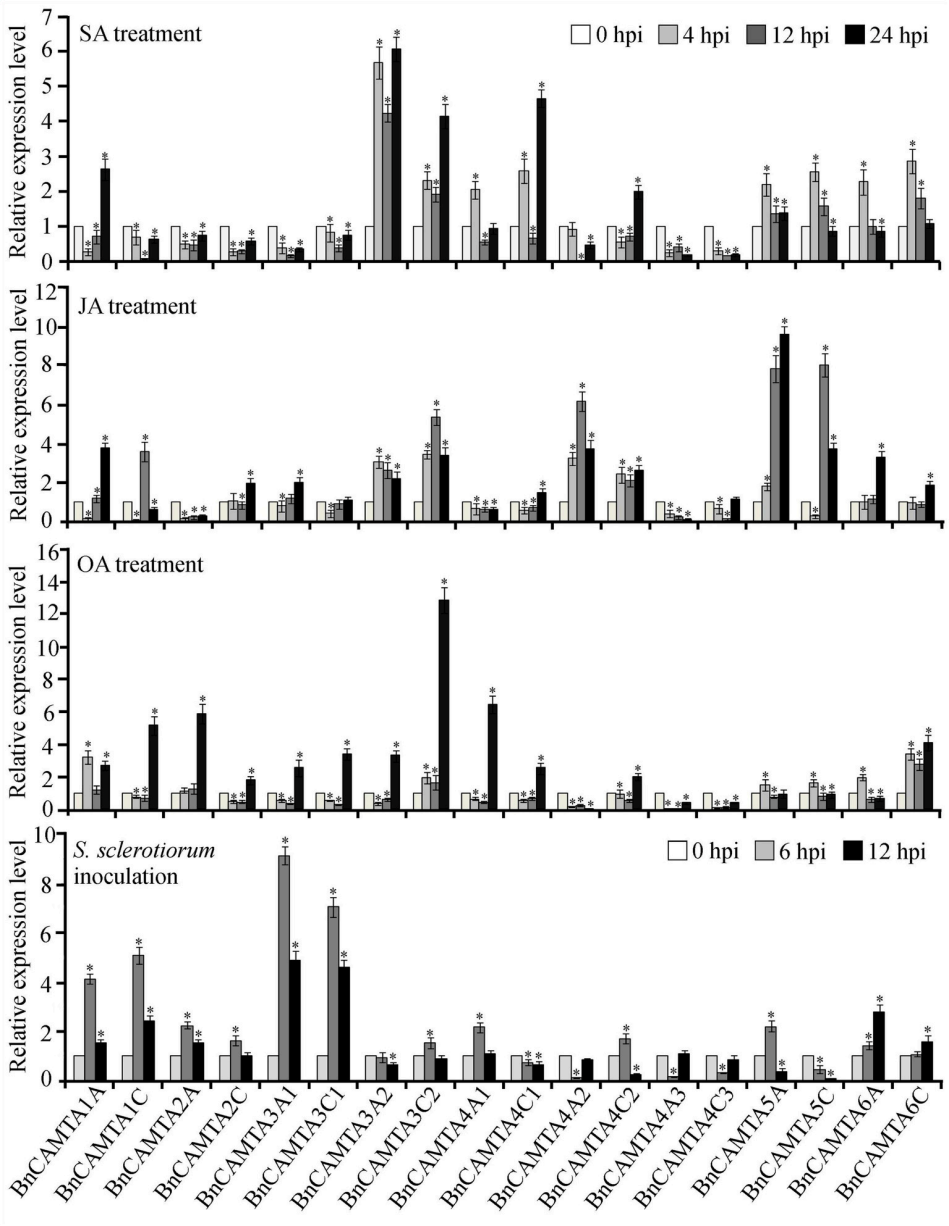
**Expression profiles of *BnCAMTA* genes in response to SA, JA and OA treatments and *S*. *sclerotiorum* inoculation**. SA (salicylic acid, 1 mM), JA (jasmonic acid, 200 μM), and OA (oxalic acod, 1 mM)-sprayed and *S. sclerotiorum-*inoculated leaves of 4-week-old oilseed rape plants were sampled at the indicated time points for qRT-PCR analyses with β -Tubulin gene serving as a loading control gene. Data represent the mean ± SE of three independent experiments. Significant difference between mean values is indicated as a “*” (*p* < 0.05, DMRT).

To obtain a clue on functions of *BnCAMTA* genes, expressional response of these genes to multifunctional hormones SA and JA as well as oxalic acid (OA), the toxin secreted by the pathogen *S. sclerotiorum* was detected by RT-qPCR. From treatment perspective, SA strongly induced expression of *BnCAMTA*s 3A2, 3C2, and 4C1 by over 4 folds in at least one time point, and moderately induced expression of *BnCAMTA*s 4A1, all 5s and 6s by around 2 folds at the early time point (4 hpi) as well as that of *BnCAMTA*s 1A and 4C2 at the late time point (24 hpi), but repressed expression of the remaining *BnCAMTA* genes. JA strongly induced expression of *BnCAMTA*s 3C2, 4A2 and all two 5s by over 4 folds at 12 hpi, and moderately induced expression of *BnCAMTA*s 1A, 1C, 2C, 3A1, 3A2, 4C2, and 6s at 12 and/or 24 hpi, but suppressed expression of the remaining *BnCAMTA* genes. OA generally induced expression of *BnCAMTA* genes at 24 hpi however, repressed expression of three subgroup 4 *BnCAMTA* genes (4A2, 4A3, and 4C3; Figure 5). From gene perspective, expression of most of the *BnCAMTA* genes was up-regulated by these three stimuli, although the level of alteration varied in response to different stimulus. However, expression of *BnCAMTA* genes 4A3 and 4C3 was significantly down-regulated by all stimuli. In addition, expression of *BnCAMTA* genes 1C, 2A, 2C, 3A1, 3C1, and 4A2 was reduced by SA, while expression of *BnCAMTA2A* and *BnCAMTA4A2* was repressed by JA and OA, respectively (Figure 5). The results indicated that the *BnCAMTA* genes widely but differentially respond at expression level to the three defense and stress-related signaling molecules SA, JA and OA.

### Expression of *BnCAMTA* genes during the early phase of *S. sclerotiorum* infection

To probe the potential roles of BnCAMTAs in resistance to *S. sclerotiorum*, expression of the 18 *BnCAMTA* genes in oilseed rape leaves after *S. sclerotiorum* inoculation was inspected by qRT-PCR. The result showed that expression of six *BnCAMTA* genes (1A, 1C, 2A, 3A1, 3C1, and 6A) was significantly increased by over 2 folds after pathogen inoculation, peaking at 6 hpi except *BnCAMTA6A*, which reached a maximum at 12 hpi (Figure 5). Among them, *BnCAMTA3A1* and *BnCAMTA3C1* exhibited the most drastic change in expression. Their transcripts were increased by 9.1 and 7.0 folds, respectively, at 6 hpi. In addition, expression of six other *BnCAMTA* genes (2C, 3C2, 4A1, 4C2, 5A, and 6C) was also up-regulated in at least one time points but only at a change fold of less than 2.0. On the contrary, expression of four subgroup 4 *BnCAMTA* genes (4A2, 4A3, 4C1, and 4C3) and *BnCAMTA5C* was strongly decreased at the early time point of pathogen inoculation (6 hpi; Figure 5). These results confirmed that *BnCAMTA* genes are differentially transcriptionally responsive to *S. sclerotiorum* infection at the early phase.

### Exogenous supply of SA, JA, and OA altered resistance against *S. sclerotiorum* in oilseed rape plants

**Figure 6:**
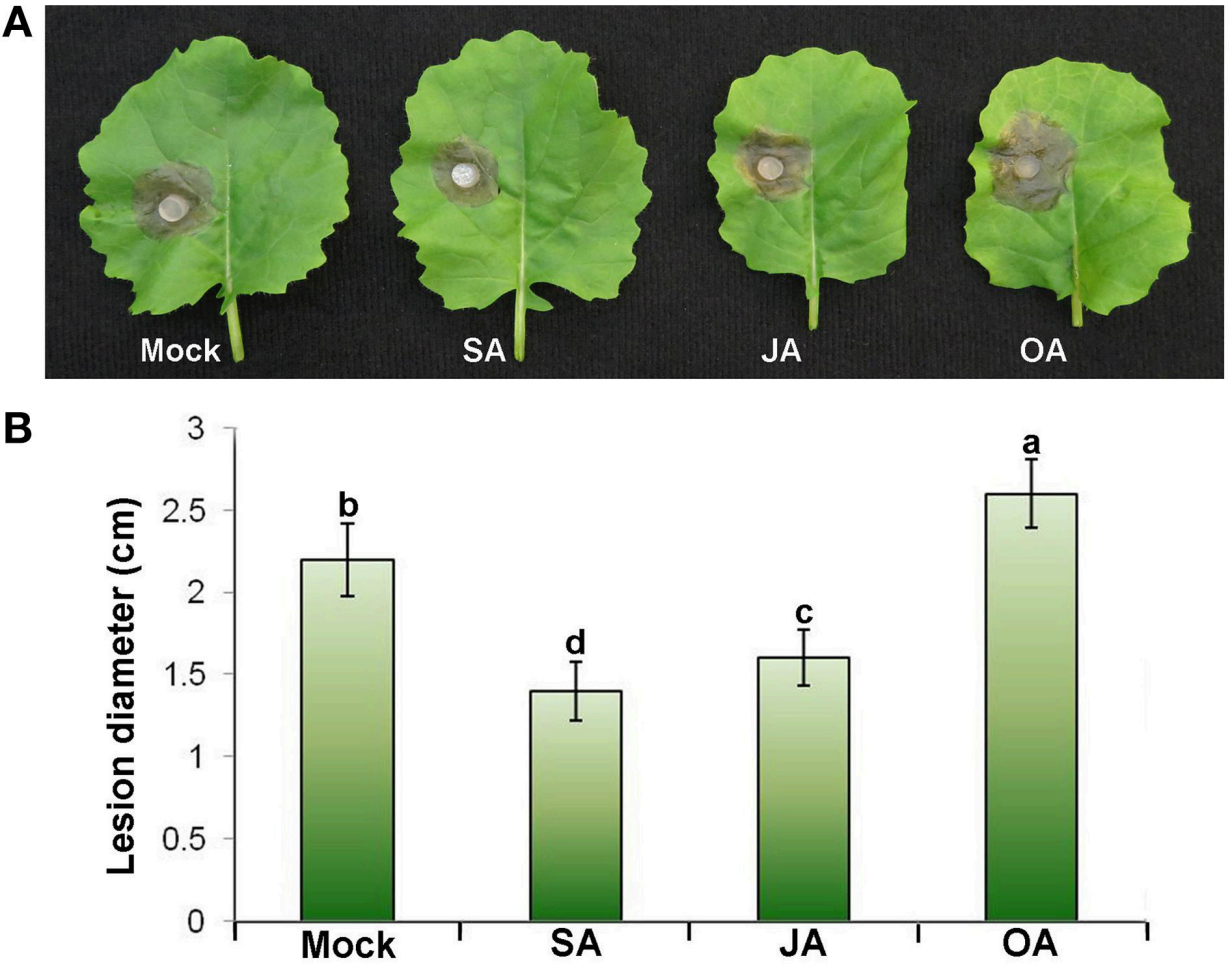
**Effect of exogenous treatment with SA, JA, and OA on resistance against *S. sclerotiorum* in oilseed rape plants. (A)** The necrotic disease symptoms caused by *S. sclerotiorum* inoculation in leaves pretreated with 0.01% ethanol (mock), SA (1 mM), JA (200 μM), and OA (1 mM), respectively, at 4 h prior to pathogen inoculation. The photographs were taken at 36 hpi. **(B)** Statistical analysis of the lesion diameter. Data represent the mean ± SE of three independent experiments. Significant difference between mean values is indicated as small letters (*p* < 0.05, DMRT).

The observation that some *BnCAMTA* genes are strongly responsive to SA, JA, and OA treatments and *S. sclerotiorum* inoculation prompted us to investigate the effect of these chemicals on resistance to *S. sclerotiorum* in oilseed rape plants. Leaves of oilseed rape plants were treated with these chemicals and inoculated with *S. sclerotiorum* at 4 h after treatments. As shown in Figure 6, SA and JA treatments obviously enhanced oilseed rape resistance to *S. sclerotiorum*, but OA treatment reduced plant resistance, as manifested by that *S. sclerotiorum* caused necrotic lesions were significantly smaller (1.4 and 1.6 cm at diameter) in SA- and JA-treated leaves, but larger (2.6 cm at diameter) in OA-treated leaves, than those (2.2 cm at diameter) in mock-inoculated control leaves at 36 hpi (Figure 6). This result indicated that SA and JA are associated with oilseed rape resistance to *S. sclerotiorum*.

### Arabidopsis *CAMTA3* negatively regulated resistance to *S. sclerotiorum*

**Figure 7:**
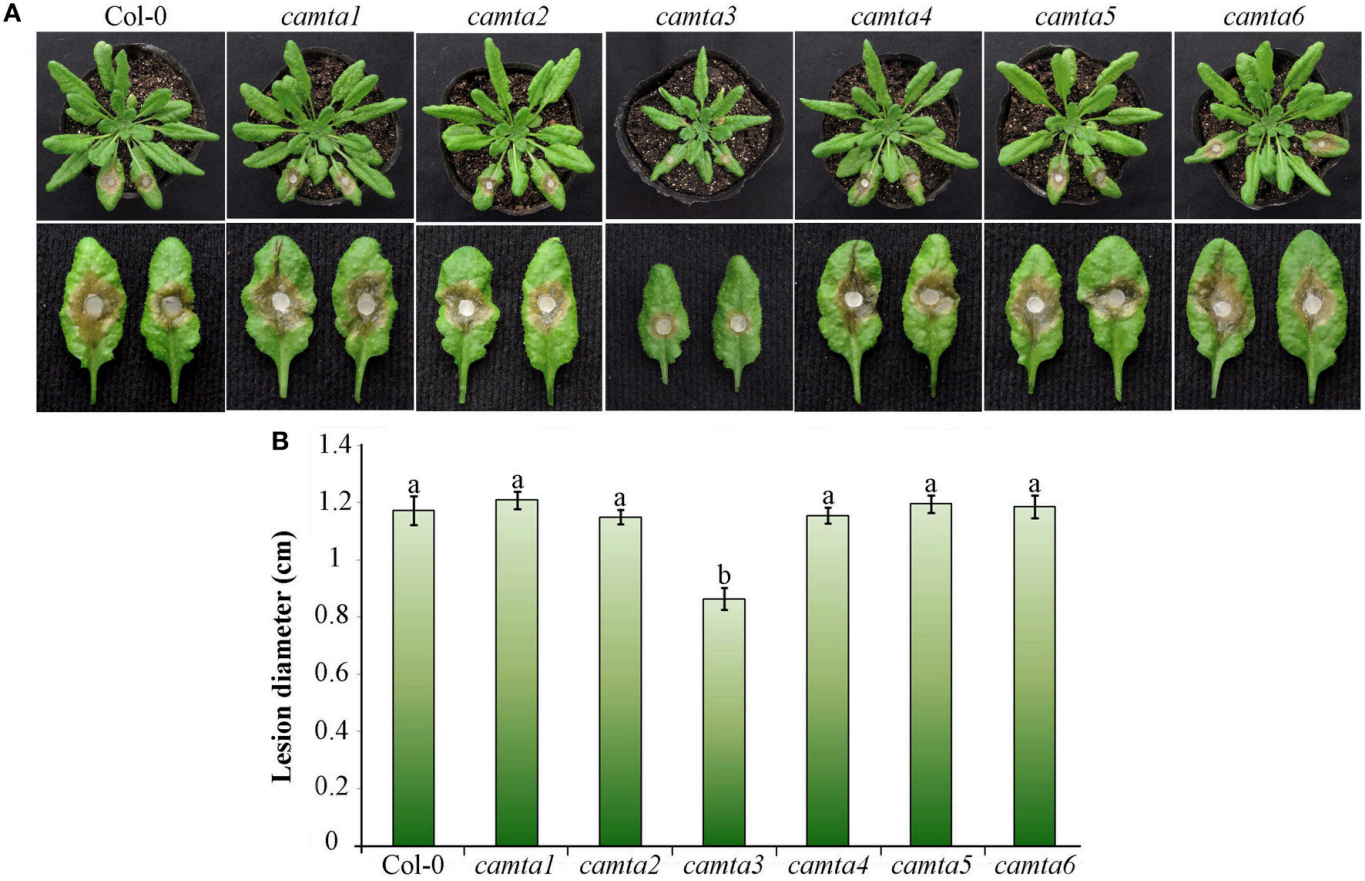
***Atcamta3* mutant plants exhibited enhanced resistant to *S. sclerotiorum***. **(A)** The necrotic disease symptoms of the *Atcamta* mutants and Col-0 wild-type plants (upper panel) and detached leaves (lower panel) after inoculated with *S. sclerotiorum*. Photographs were taken at 30 hpi. **(B)** Statistical analysis of the lesion diameter. Data represent the mean ± SE of three independent experiments. Significant difference between mean values is indicated as small letters (*p* < 0.05, DMRT).

To further explore the role of *CAMTA*s in plant resistance, we preformed inoculation analyses in six *Atcamta* mutants to examine their response to the devastating necrotrophic fungal pathogen *S. sclerotiorum*. Results of the inoculation analyses showed that the *S. sclerotiorum* caused necrotic lesions in the *camta3* plants were significantly smaller (0.86 cm at diameter) than those in wild-type and the other mutant plants (over 1.17 cm at diameter) at 24 hpi (Figure 7), demonstrating that the *camta3* mutant plants were more resistant to *S. sclerotiorum* in comparison with wild-type and the other *camta* mutant plants. This result revealed that *CAMTA3* plays a negative role in plant resistance to *S. sclerotiorum*.

### Arabidopsis *CAMTA3* negatively regulated chitin-elicited accumulation of hydrogen peroxide

**Figure 8:**
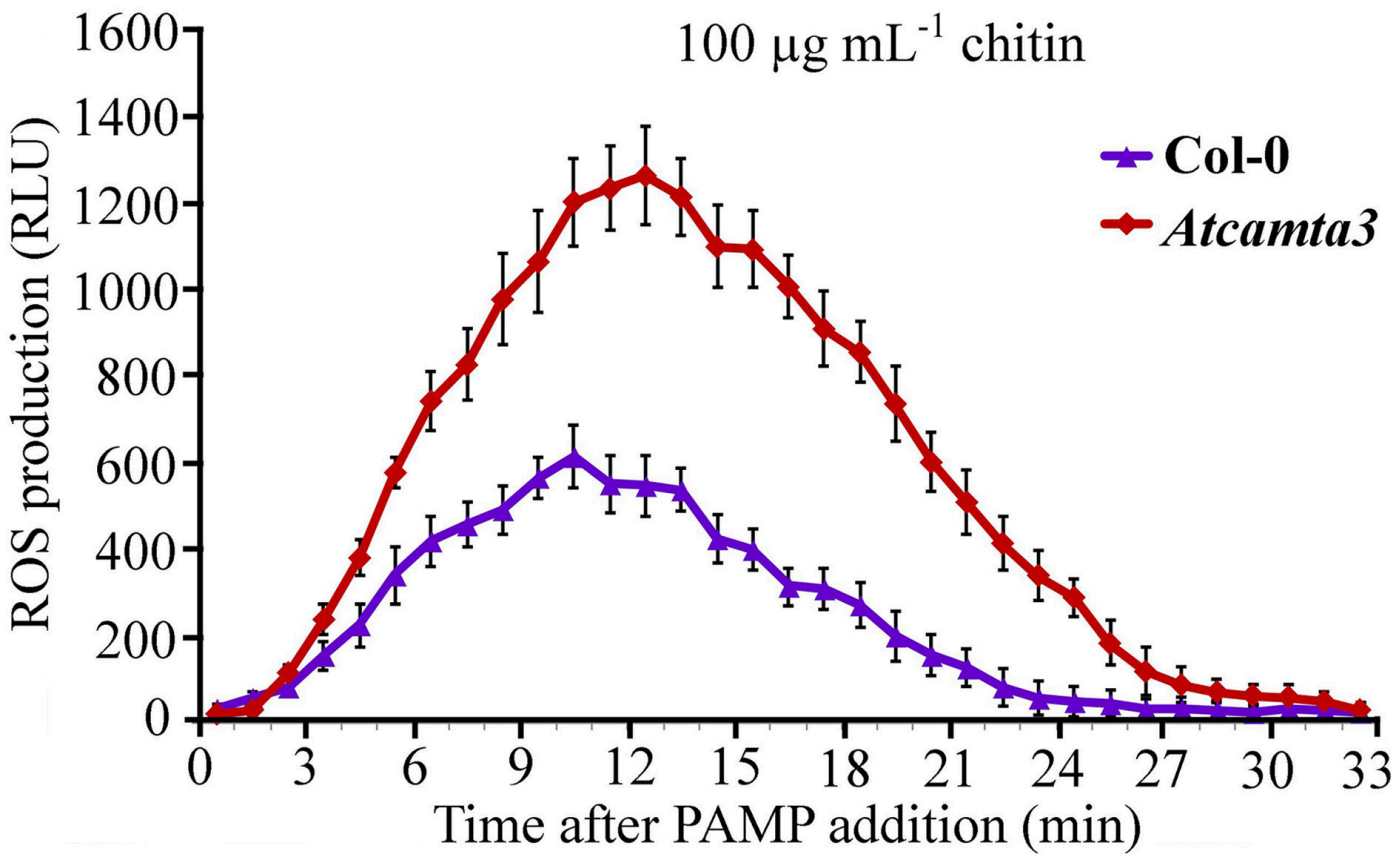
***Atcamta3* mutant plants accumulated higher level of chitin-elicited H_2_O_2_**. Dynamics of H_2_O_2_ accumulation in response to chitin elicitation in leaves of Col-0 and *Atcamta3* mutant plants was shown. Chitin-triggered H_2_O_2_ bursts were measured using luminol-based assay in leaf discs of Col-0 and *Atcamta3* mutant plants. Data are shown as relative luminal units (RLU) and represent the mean ± SE of three independent experiments.

To provide some insights into the mechanisms of *AtCAMTA3* to regulate plant resistance, we inspected effect of AtCAMTA3 on accumulation of hydrogen peroxide induced by the PAMP chitin, which exists in the cell wall of the fungal pathogen *S. sclerotiorum*. In response to 100 μg mL^−1^ chitin, *atcamta3* mutant plants accumulated much higher level of hydrogen peroxide, peaking at over 1200 RLU, than wild-type plants, culminating at about 600 RLU under current measuring system (Figure 8). This result demonstrated that *AtCAMTA3* negatively regulates chitin-triggered PTI as manifested by its negative regulation on chitin-triggered accumulation of hydrogen peroxide.

### Arabidopsis *CAMTA3* negatively regulated the expression of a set of CGCG-Box containing defense signaling genes

**Figure 9:**
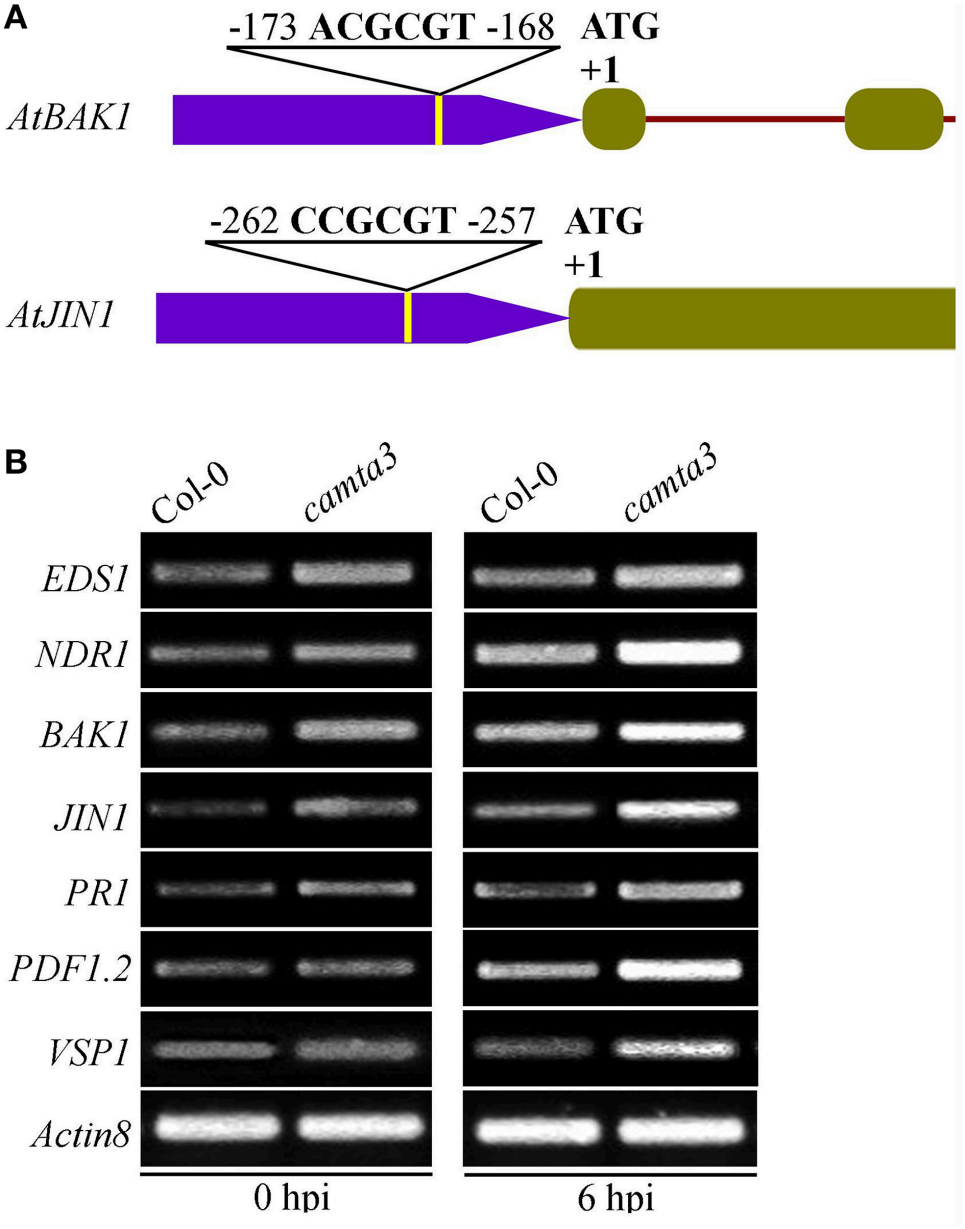
**Expression patterns of AtCAMTA3 target genes *EDS1* and *NDR1* and putative target genes *BAK1* and *JIN1* in *Atcamta3* mutant plants. (A)** Schematic representation of CGCG elements in promoter regions of the *BAK1* and *JIN1* genes. **(B)** Expression patterns of four confirmed or putative AtCAMTA3-targeted genes and three defense signaling pathway marker genes (*PR1, PDF1.2*, and *VSP1*) in Col-0 wild-type and *Atcamta3* mutant plants at 0 and 6 h post *S. sclerotiorum* inoculation. Gene expression was examined by semiquantitative RT-PCR with the Arabidopsis ACTIN8 gene serving as a loading control gene. The profile of electrophoresis on a 1.5% agarose gel of the products obtained from 32 cycles (28 cycles for control) of PCR was shown.

To further elucidate the mechanisms of *AtCAMTA3* in regulating resistance to *S. sclerotiorum*, we examined the expression of four putative or confirmed *AtCAMTA3* targeted genes (*EDS1, NDR1, BAK1*, and *JIN1*) and three defense signaling pathway marker genes (*PR1, PDF1.2*, and *VSP1*) in wild-type and *Atcamta3* mutant plants before and after inoculating with *S. sclerotiorum*. These four genes were selected for this study because they are known to play important roles in plant resistance and PTI to *S. sclerotiorum* and/or other pathogens (Guo and Stotz, 2007; Du et al., 2009; Perchepied et al., 2010; Nie et al., 2012; Zhang et al., 2013; Macho and Zipfel, 2014). Meanwhile, *EDS1* and *NDR1* are targets of AtCAMTA3 (Du et al., 2009; Nie et al., 2012). Here, we found that the two PTI and/or *S. sclerotiorum* resistance regulatory genes *BAK1* and *JIN1* also contained a CGCG *cis*-element in the region of –173 to –168 (ACGCGT) and –262 to –257 (CCGCGT), respectively, of their promoters (Figure 9A), they are therefore the potential targets of CAMTA3. Semiquantitative RT-PCR analysis revealed that the expression of *EDS1, NDR1, BAK1*, and *JIN1* in *Atcamta3* plants was obviously increased compared with the wild-type plants (Figure 9B), demonstrating that *AtCAMTA3* negatively regulates chitin-triggered immunity and resistance to *S. sclerotiorum* probably via negatively and directly regulating the expression of *EDS1, NDR1, BAK1*, and *JIN1*. Moreover, expression of *PR1, PDF1.2*, and *VSP1*, marker genes of SA, ethylene and JA defense signaling pathways was obviously higher in *Atcamta3* plants than in wild type plants (Figure 9B), indicating that *AtCAMTA3* negatively regulates resistance to *S. sclerotiorum* probably through modulating SA, ethylene and JA defense signaling pathways. In addition, in *Atcamta3* plants, transcripts of *EDS1, NDR1, BAK1*, and *JIN1* were still increased in response to *S. sclerotiorum* inoculation at 6 hpi (Figure 9B), suggesting that factor(s) other than AtCAMTA3 might respond to *S. sclerotiorum* inoculation to promote the expression of these defense signaling genes in *Atcamta3* plants.

## Discussion

### Composition and functions of *CAMTA* gene family in oilseed rape

In this study, we found that oilseed rape possesses a total of 18 CAMTAs. Oilseed rape is thus the plant species containing the highest number of CAMTAs among over 40 plant species whose *CAMTA* family has been identified to date (Bouché et al., 2002; Choi et al., 2005; Koo et al., 2009; Yang et al., 2012; Shangguan et al., 2014; Wang et al., 2015; Yang et al., 2015; Yue et al., 2015; Rahman et al., 2016). The number of *CAMTA* genes in oilseed rape is 3 folds as many as that in Arabidopsis, which is consistent with the ratio of total number of transcription factors in oilseed rape to that in Arabidopsis (Chalhoub et al., 2014). An important reason that oilseed rape carries so high number of CAMTAs is that oilseed rape is a tetraploid of the two progenitors *B. rapa* and *B. oleracea*, and thus contains copies of genes from both progenitors. As a matter of fact, we found that *B. rapa* and *B. oleracea* each own 9 CAMTAs, while the oilseed rape subgenomes A and C, which correspond to genomes of *B. rapa* and *B. oleracea*, respectively, each contain 9 CAMTAs with identical composition of subgroups as observed as *B. rapa* and *B. oleracea* (Table 1, Table S1, and Figure 3). Another reason that oilseed rape carries much higher number of CAMTAs than Arabidopsis is that oilseed rape genome has undergone *CAMTA* gene expansion compared with Arabidopsis genome. The subgenomes A and C of oilseed rape and genomes of *B. rapa* and *B. oleracea*, each contain 9 CAMTAs with 2 CAMTA3s and 3 CAMTA4s (Table 1, Table S1, and Figure 3), demonstrating that genomes of all three *Brassica* species have expanded *CAMTA3* and *CAMTA4* genes and this expansion in oilseed rape occurred before formation of oilseed rape. Expansion of *CAMTA3* and *CAMTA4* genes in *Brassica* species is unique in all 11 species belonging to *Brassicaceae, Caricaece, Malvaceae, Rutaceae*, and *Myrtaceae* of Rosids whose *CAMTA* family has been identified to date (Figures 1, 2 in Rahman et al., 2016). The reason and significance of this expansion are unclear. While the function of CAMTA4 remains unknown, CAMTA3 in Arabidopsis has been well-recognized to play important roles in host and nonhost resistance against various pathogens (Galon et al., 2008; Du et al., 2009; Nie et al., 2012; Rahman et al., 2016). Therefore, whether both members of CAMTA3 in *B. rapa* and *B. oleracea* and all four in oilseed rape function in disease resistance is worthy of experimental clarification. In view of our comprehensive expression analyses, different members of subgroups BnCAMTA3 and BnCAMTA4 exhibit distinct expression profiles both constitutively in various tissues and in response to hormone treatments and pathogen inoculation. *BnCAMTA3A1* and *BnCAMTA3C1* are highly expressed in stem, cotyledon and true leaf while *BnCAMTA3A2* and *BnCAMTA3C2* are nearly not expressed in all these tissues (Figure 4). Meanwhile, *BnCAMTA3A1* and *BnCAMTA3C1* are not obviously responsive to SA and JA treatments but strongly responsive to *S. sclerotiorum* inoculation, while conversely, *BnCAMTA3A2* and *BnCAMTA3C2* are highly responsive to SA and JA treatments but not significantly responsive to *S. sclerotiorum* inoculation (Figure 5). Moreover, the gene structure of *BnCAMTA3C2* is distinct to the other members of *BnCAMTA3* subgroup (Table 1; Figure 1B). Similarly, distict expression patterns both constitutively in various tissues and in response to hormone treatments and pathogen inoculation are also observed for different members of *BnCAMTA4* genes (Figures 4, 5). The gene structure of *BnCAMTA4A2* and the pI value of *BnCAMTA4A2* and *BnCAMTA4C2* are distinguishable from the other members of *BnCAMTA4* subgroup (Table 1; Figure 1B). Therefore, different members of subgroups BnCAMTA3 and BnCAMTA4 are most likely to play different roles in development, abiotic stress tolerance, and disease resistance. This seems to be also the case for functions of different subgroups of the *CAMTA* gene family in oilseed rape considering their distinct expression profiles both constitutively in various tissues and in response to diverse abiotic and biotic stimuli.

### Role and mechanism of *AtCAMTA3* in PTI and resistance to the necrotrophic pathogen *S. sclerotiorum*

Role of CAMTAs in disease resistance against a wide range of biotrophic pathogens in various plants has been reported. These pathosystems include Arabidopsis against bacterial pathogens *Pst* DC3000 (Du et al., 2009) and *Xoo* (Rahman et al., 2016) as well as the fungal pathogen *Golovinomyces cichoracearum* (Nie et al., 2012), and rice against the bacterial pathogen *Xoo* and the fungal pathogen *Magnaporthe grisea* (Koo et al., 2009). However, Function of CAMTAs in plant disease resistance against necrotrophic pathogens has only been reported for one pathogen *Botrytis cinerea* (Galon et al., 2008; Li et al., 2014). In this study, using *camta* mutants, we demonstrate that AtCAMTA3 negatively regulates the resistance to the typical necrotrophic pathogen *S. sclerotiorum*, which is one of the most devastating fungal pathogens and causes the most imprtant disease, the white mold disease, in one of the most important oil-producing crops oilseed rape (Bolton et al., 2006). Additionally, oilseed rape *CAMTA* genes 1A, 1C, 3A1, and 3C1 are strongly responsive to *S. sclerotiorum* inoculation but differentially respond to the treatment with SA and JA, which play important roles in resistance to *S. sclerotiorum* (Guo and Stotz, 2007; Perchepied et al., 2010). Therefore, these four *CAMTA* genes may also play a role in resistance to *S. sclerotiorum* in oilseed rape. Taken together, these studies reveal that *CAMTA* genes, especially *CAMTA3*, contribute greatly to resistance against both biotrophic and necrotrophic pathogens in various plant species.

In addition, in this study, we provide some new intriguing points for the mechanisms of CAMTA3 to regulate PTI and *S. sclerotiorum* resistance. First, BAK1 might be the target of CAMTA3. BAK1 is a pivotal receptor kinase in PTI triggered by diverse PAMPs such as bacterial PAMP fig22 and fungal PAMP chitin (Macho and Zipfel, 2014). More importantly, it is also required for PTI triggered by SCFE1, a putative PAMP purified from *S. sclerotiorum* (Zhang et al., 2013). Interestingly, we found in this study that the *AtBAK1* gene contains a CGCG *cis*-element in the region of –173 to –168 (ACGCGT) of its promoter (Figure 9A). Furthermore, expression of *AtBAK1* is greatly enhanced in *Atcamta3* mutant plants compared with wild-type plants (Figure 9B). Moreover, *Atcamta3* mutant plants accumulate much higher level of chitin-elicited hydrogen peroxide than wild-type plants (Figure 8). Collectively, our results indicate that AtCAMTA3 negatively regulates the resistance to *S. sclerotiorum* probably via suppressing *AtBAK1*-meadited PTI. Second, CAMTA3 may target JIN1/MYC2 to directly modulate JA signaling thereby regulating plant defense against pathogens including *S. sclerotiorum*. JA signaling pathway is one of the most important plant defense pathways. This pathway is essential to the reisitance to *S. sclerotiorum* (Guo and Stotz, 2007; Perchepied et al., 2010). As a key component of JA signaling pathway, JIN1 is indispensible for the resistance to *S. sclerotiorum* (Guo and Stotz, 2007). We found that the *JIN1* gene contains a CGCG *cis*-element in the region of −262 to −257 (CCGCGT) of its promoter (Figure 9A). Further, expression of *AtJIN1* is greatly enhanced in *Atcamta3* mutant plants than in wild-type plants (Figure 9B). Together, our results suggest that AtCAMTA3 may modulate the JA signaling pathway via direct targeting JIN1 and thereby regulates the resistance to pathogens including *S. sclerotiorum*. In these scenarios, it will be very intriguing to confirm whether CAMTA3 can indeed direct bind and regulate expression of *BAK1* and *JIN1* by other approaches such as ChIP and EMSA assays. Finally, we oberved that expression of CAMTA3-targeted *EDS1* and *NDR1* genes is obviously increased in *Atcamta3* mutant plants than in wild-type plants (Figure 9B) as reported previously (Du et al., 2009; Nie et al., 2012; Rahman et al., 2016). These genes act upstream of SA signaling, which play a role in resistance to *S. sclerotiorum* (Guo and Stotz, 2007). Thus, *EDS1* and *NDR1* genes may also contribute to this resistance. The confirmation of function of these genes in this resistance will clarify the significance of CAMTA3-targeting of these two genes in resistance to *S. sclerotiorum*.

**Figure 10:**
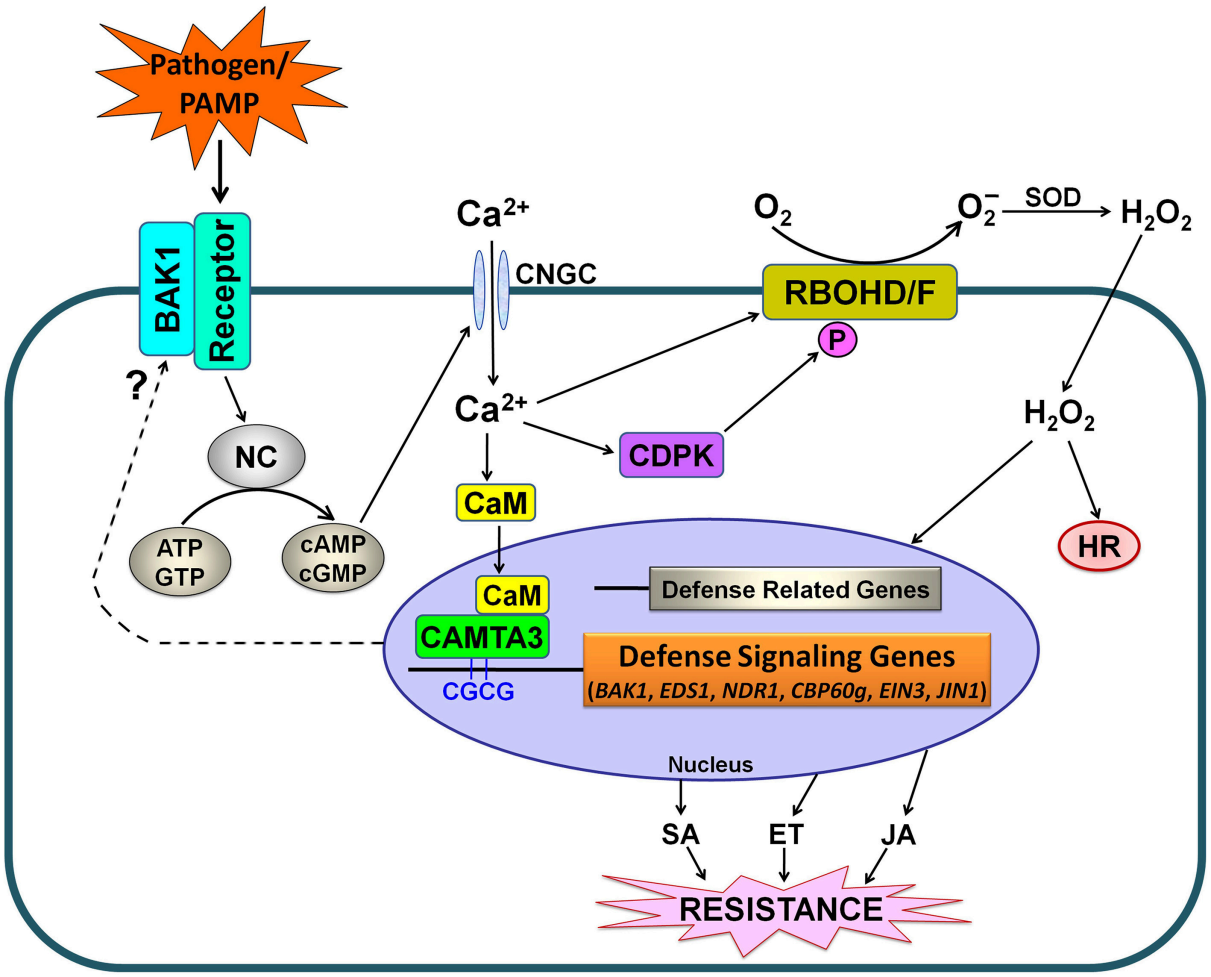
**Simplified schematic work model for CAMTA3-mediated defense signaling in plants**. PAMPs from pathogens or exogenously supply are recognized by plant receptor proteins. Activation of the recognition complex generally requires BAK1. This recognition may lead to the activation of NC and consequently a rise in the concentration of cyclic nucleotides (cAMP and cGMP), resulting in cytosolic Ca^2+^ influx through opening of Ca^2+^ channels such as CNGCs. The cytosolic Ca^2+^ transients modulate CDPK-promoted and RBOHD/F-mediated ROS accumulation as well as CAMTA3-mediated denfense signaling. The activated CAMTA3 directly binds to the CGCG *cis*-elements in the promoter of defense-related target genes including *EDS1, NDR1, CBP60g, EIN3*, and *JIN1* and regulate their expression, which modulates the accumulation and signaling of SA, ET and JA, and thereby alters disease resistance. Additionally, CAMTA3 may target *BAK1* to mudulate the recognition complex, reflecting its global control of plant defense through regulating expression of the target genes at multiple nodes of the defense network. Abbreviations: BAK1, BRI1-associated receptor kinase; CaM, calmodulin; *CAMTA*, calmodulin binding transcriptional activator; CDPK, calcium-dependent protein kinases; CNGC, cyclic nucleotide gated channel; ATP, adenosine triphosphate; GTP, guanosine triphosphate; cAMP, cyclic adenosine monophosphate; cGMP, cyclic guanosine monophosphate; NC, nucleotidyl cyclase; HR, hypersensitive response; SOD, superoxide dismutase; SA, salicylic acid; ET, ethylene; JA, jasmonic acid.

Based on our findings and the previously published reports (Benn et al., 2014; Rahman et al., 2016), we propose a schematic model for CAMTA3-mediated signaling in plants in response to pathogens and PAMPs (Figure 10). In this model, stimuli including pathogens such as *S. sclerotiorum* and *Xoo* as well as PAMPs such as chitin and flg22, may activate nucleotidyl cyclase (NC) to generate cyclic nucleotides including cyclic adenosine monophosphate (cAMP) and cyclic guanosine monophosphate (cGMP), which activate Ca^2+^ channels such as cyclic nucleotide gated channels (CNGCs), leading to cytosolic Ca^2+^ influx (Qi et al., 2010; Ma and Berkowitz, 2011; Saand et al., 2015a,b). The cytosolic Ca^2+^ elevations are transduced by various Ca^2+^ sensor proteins including CaM, which activates CAMTA3. The activated CAMTA3 directly binds to the CGCG *cis*-elements in the promoter of defense-related target genes including *EDS1, NDR1, CBP60g, EIN3*, and *JIN1* and regulate their expression, which modulates the accumulation and signaling of SA, ET, and JA, and thereby alters disease resistance. Simultaneously, increased cytosolic Ca^2+^ would activate calcium-dependent protein kinases (CDPKs), which subsequently phosphorylate and activate RBOHD/F, resulting in ROS accumulation and thereby affecting hypersensitive response (HR) and plant disease resistance. Intriguingly, CAMTA3 may target *BAK1* to modulate the recognition complex, a beginning step for plant response to pathogens and PAMPs (Figure 10).

## Conclusion

In the present study, we have identified and characterized 18 *CAMTA* genes in oilseed rape genome. They were inherited from the nine copies each in its progenitors *B. rapa* and *B. oleracea* and represented the highest number of CAMTAs in a given plant species identified to date. The oilseed rape CAMTAs clustered into three major groups and had expanded subgroups CAMTA3 and CAMTA4 uniquely in rosids species, which occurred before formation of oilseed rape. Comprehensive expression analyses indicated that *BnCAMTA* genes are likely to play distinct roles in development, abiotic stress tolerance and disease resistance. Among the four *BnCAMTA3* genes, *BnCAMTA3A1* and *BnCAMTA3C1* are most probably the functional homologs of *AtCAMTA3* and contribute to plant defense. Furthermore, functional analyses employing Arabidopsis *camta* mutants revealed that *CAMTA3* negatively regulates PAMP triggered immunity (PTI) probably by directly targeting *BAK1* and it also negatively regulates plant defense against pathogens such as *S. sclerotiorum* through suppressing JA signaling pathway probably via directly targeting *JIN1*. Our findings provide some insights into the composition of CAMTAs and their roles and functional mechanisms in plant defense.

## Author contributions

HR and XZ conducted the bioinformatics and phylogenetic analyses. HR and YX carried out the gene expression and functional analysis, designed and analyzed all statistical data. XC conceived of the study, and participated in its design and coordination. XC and HR prepared the manuscript.

### Conflict of interest statement

The authors declare that the research was conducted in the absence of any commercial or financial relationships that could be construed as a potential conflict of interest.
