# Supplementary material for: Brassica napus Genome Possesses Extraordinary High Number of CAMTA Genes and CAMTA3 Contributes to PAMP Triggered Immunity and Resistance to Sclerotinia sclerotiorum
Source: Front Plant Sci. 2016 May 4;7:581. doi: 10.3389/fpls.2016.00581 (PMC4854897; doi:10.3389/fpls.2016.00581)
Supplement: Supplementary file 1 [file Table1.PDF]

Supplementary Material

Brassica napus genome possesses extraordinary high number of *CAMTA* genes and *CAMTA3* contributes to PAMP triggered immunity and resistance to *Sclerotinia sclerotiorum*

Hafizur Rahman[1], You-Ping Xu[2], Xuan-Rui Zhang[1], Xin-Zhong Cai[1*]

[1] Institute of Biotechnology, College of Agriculture and Biotechnology, Zhejiang University, Hangzhou, China

[2] Center of Analysis and Measurement, Zhejiang University, Hangzhou, China

***Corresponding author:** Xin-Zhong Cai, Institute of Biotechnology, College of Agriculture and Biotechnology, Zhejiang University, 866 Yu Hang Tang Road, Hangzhou 310058, China.
E-mail: xzhcai@zju.edu.cn

Supplementary Tables

Table S1 | RT-PCR primers used in this study.

Gene	Primer Name	Sequence (5'-3')
BnCAMTA1A	BnCAMTA1a-F	AACGGAGAAAGAAAATATCGG
	BnCAMTA1a-R	GACTAATAAATCCAAAAGATCCA
BnCAMTA1C	BnCAMTA1b-F	CAGTTAACACAAAGAAGCTTC
	BnCAMTA1b-R	GCTAATAAATCCAAAAGATCCGC
BnCAMTA2A	BnCAMTA2a-F	ACAACAATAGCAATACAGAGG
	BnCAMTA2a-R	AGCAAAACCTAAATGAAGTTAGAA
BnCAMTA2C	BnCAMTA2b-F	TTCATACATACCCAGATCGAT
	BnCAMTA2b-R	GAATATCTAATTGAGGGGCAAAT
BnCAMTA3A1	BnCAMTA3a-F	AGAAAGCTTTCAACTGTTCCTA
	BnCAMTA3a-R	CCAAAACGTCTTGCTTCCGC
BnCAMTA3C1	BnCAMTA3b-F	GAAGAAAGCTTTCACTTTTACTG
	BnCAMTA3b-R	CTAAATCGTCTTGCTTCCGC
BnCAMTA3A2	BnCAMTA3d-F	TGCGTCAATGAGGAATTTGAC
	BnCAMTA3d-R	GAAACTTTTGGTAGTTCTGTAAGA
BnCAMTA3C2	BnCAMTA3c-F	AAGAAACAAAAGTCTATTGATTC
	BnCAMTA3c-R	TAAACCTTCAGCTCCAGGGC
BnCAMTA4A1	BnCAMTA4d-F	TTCCTCCTCCTTGTTATTCTG
	BnCAMTA4d-R	AAGGAACTTGAGCGTTTCCAA
BnCAMTA4C1	BnCAMTA4c-F	GATTATGAGGATGATGATGCCT
	BnCAMTA4c-R	TACTAACAACAAGCTTCTCCT
BnCAMTA4A2	BnCAMTA4f-F	GAGAAAGTGCAACACTCCAAA
	BnCAMTA4f-R	GGAGATTCGTTAGACTGATTC
BnCAMTA4C2	BnCAMTA4e-F	CGGATCTCTTTTCAAGTCCAGT
	BnCAMTA4e-R	CCGTTACTCTTAAAGACAACGT
BnCAMTA4A3	BnCAMTA4b-F	ATGAGTAGCACCACAATTTTGG
	BnCAMTA4b-R	ACTGGGAGGGTTTTGAGGTG
BnCAMTA4C3	BnCAMTA4a-F	ATGGCTTCACATTTAAAAACC
	BnCAMTA4a-R	GTAGGGTTTTGAGGTGGCGT
BnCAMTA5A	BnCAMTA5b-F	TAATCAGATGGATATTGAGAG
	BnCAMTA5b-R	ATGACAAGACGCCTCTAAGC
BnCAMTA5C	BnCAMTA5a-F	TGATCAGATGGATATGGAGAG
	BnCAMTA5a-R	ATGACAAGACGCCTCTCAGC

BnCAMTA6A	BnCAMTA6a-F	GTAGTGCGGGTCCAAGCCA
	BnCAMTA6a-R	CTCAATAGCCACCATTTTCTAA
BnCAMTA6C	BnCAMTA6b-F	ATATTTGATAGAAGTGATGGCT
	BnCAMTA6b-R	CAACATATATTGGCTTAGTTATGT
Bnβ-Tubulin	Bnβ-Tubulin-F	AGGTCTCCGACACTGTTGTTG
	Bnβ-Tubulin-R	GGAGTTGAGTTGACCAGGGA
AtEDS1	AtEDS1-F	GTCTACGCTCAATGACCTTGGAGTG
	AtEDS1-R	CATTTTTATGGGCTTGACACTTTGG
AtNDR1	AtNDR1-F	CTTTTCTTATGGCTTAGTCTCCGTG
	AtNDR1-R	ATCTTGGTCGTGTTGATGGTGG
AtJIN1	AtJIN1-F	ATTACCGGCTACAACCAACG
	AtJIN1-R	AGTTGTTTCAGTCGTCGCC
AtBAK1	AtBAK1-F	CAAATTAAGTCAACGGGTGG
	AtBAK1-R	CCCTGATAAAAGGGATCTCG
AtPR1	AtPR1-F	TCATGGCTAAGTTTGCTTCC
	AtPR1-R	AATACACACGATTTAGCACC
AtPDF1.2	AtPDF1.2-F	GGTTGGAATATGGTAGTGCTGT
	AtPDF1.2-R	CTGAACGGATGAAGGTGGAA
AtVSP1	AtVSP1-F	TCTGACAGATGGAAAAAATTGTC
	AtVSP1-R	TTCGATCCGTTTGGCTTGAG
AtACTIN8	AtACTIN8-F	CGAGGCTCCTCTTAACCCAAA
	AtACTIN8-R	GGCACAGTGTGAGACACACCA

Table S2 | *CAMTA* genes in Arabidopsis, *Brassica rapa*, *B. oleracea* and tomato.

Gene name	Locus ID	Gene location	Gene length (bp)	No. of introns	No. of amino acid (aa)	Mol.Wt. (kDa)	pI
AtCAMTA1	AT5G09410	Ch 05: 2920827-2927420	6594	15	1066	121	6.35
AtCAMTA2	AT5G64220	Ch 05: 25686246-25692215	5970	12	1050	117.2	6.23
AtCAMTA3	AT2G22300	Ch 02: 9471388-9476646	5259	13	1032	116.1	5.37
AtCAMTA4	AT1G67310	Ch 01: 25198182-25203126	4945	12	1016	113	5.31
AtCAMTA5	AT4G16150	Ch 04: 9148059-9153292	5234	12	923	104.8	7.21
AtCAMTA6	AT3G16940	Ch 03: 5781775-5786280	4506	9	845	96.1	7.93
BrCAMTA1	Brara.J02385	A10: 17500314-17505289	4976	12	1007	113.5	5.9
BrCAMTA2	Brara.I00771	A09: 4319451-4325448	5998	11	999	111.7	5.98
BrCAMTA3-1	Brara.D01317	A04: 11905341-11910660	5320	11	1031	115.2	5.45
BrCAMTA3-2	Brara.I04591	A09: 39657582-39662868	5287	9	931	104.4	5.62
BrCAMTA4-1	Brara.G02581	A07: 21345227-21350331	5105	12	988	109.9	5.79
BrCAMTA4-2	Brara.G02700	A07: 22025415-22030693	5279	12	977	108.6	6.46
BrCAMTA4-3	Brara.B01713	A02: 9992581-9997467	4887	12	1015	113.2	5.51
BrCAMTA5	Brara.H00843	A08: 10548395-10553076	4682	12	919	104.5	7.46
BrCAMTA6	Brara.E02477	A05: 22120932-22125713	4782	11	853	97.1	6.94
BoCAMTA1	XP_013611843	C9: 51373550-51378567	5018	12	1007	113.3	6.01
BoCAMTA2	XP_013611035	C9: 5385838-5391667	5830	11	995	111	5.99
BoCAMTA3-1	XP_013636192	C4: 40416571-40421951	5381	11	1026	114.7	5.44
BoCAMTA3-2	XP_013636191	C4: 40416571-40421951	5381	11	1028	114.9	5.44

BoCAMTA4-1	XP_013591879	C6: 31380616-31385937	5322	12	976	108.5	5.82
BoCAMTA4-2	XP_013592349	C6:32488350-32493787	5438	12	986	109.3	6.39
BoCAMTA4-3	XP_013620826	C2: 16093087-16098277	5191	12	1013	113.2	5.55
BoCAMTA5	XP_013604099	C8: 14784877-14809116	24240	13	919	104.4	7.02
BoCAMTA6	XP_013586584	C5: 37992485-37997374	4890	11	853	96.9	6.84
SlCAMTA1	JN558810	Ch 01: 85225560-85236375	14060	13	1037	116.7	5.46
SlCAMTA2	JN566050	Ch 01: 53184734-53195240	13659	10	939	105.6	8.75
SlCAMTA3	GU170838	Ch 04: 53071230-53081628	13519	12	1097	122.6	5.68
SlCAMTA4-1	JN566047	Ch 12: 25868813-25886100	22474	11	906	101.8	6.81
SlCAMTA4-2	JN566048	Ch 05: 11602241-11617922	20386	12	950	106.7	6.14
SlCAMTA5	JN566049	Ch 01: 62496036-62529497	43500	12	920	104	7.92
SlCAMTA6	JN566051	Ch 12: 64863822-64873108	12073	12	910	103.3	7.01
