# Supplementary material for: Brassica napus Genome Possesses Extraordinary High Number of CAMTA Genes and CAMTA3 Contributes to PAMP Triggered Immunity and Resistance to Sclerotinia sclerotiorum
Source: Front Plant Sci. 2016 May 4;7:581. doi: 10.3389/fpls.2016.00581 (PMC4854897; doi:10.3389/fpls.2016.00581)
Supplement: Supplementary file 2 [file Image1.PDF]

Supplementary Material

Brassica napus genome possesses extraordinary high number of *CAMTA* genes and *CAMTA3* contributes to PAMP triggered immunity and resistance to *Sclerotinia sclerotiorum*

Hafizur Rahman[1], You-Ping Xu[2], Xuan-Rui Zhang[1], Xin-Zhong Cai[1*]

[1] Institute of Biotechnology, College of Agriculture and Biotechnology, Zhejiang University, Hangzhou, China

[2] Center of Analysis and Measurement, Zhejiang University, Hangzhou, China

***Corresponding author:** Xin-Zhong Cai, Institute of Biotechnology, College of Agriculture and Biotechnology, Zhejiang University, 866 Yu Hang Tang Road, Hangzhou 310058, China.
E-mail: xzhcai@zju.edu.cn

Supplementary Figures

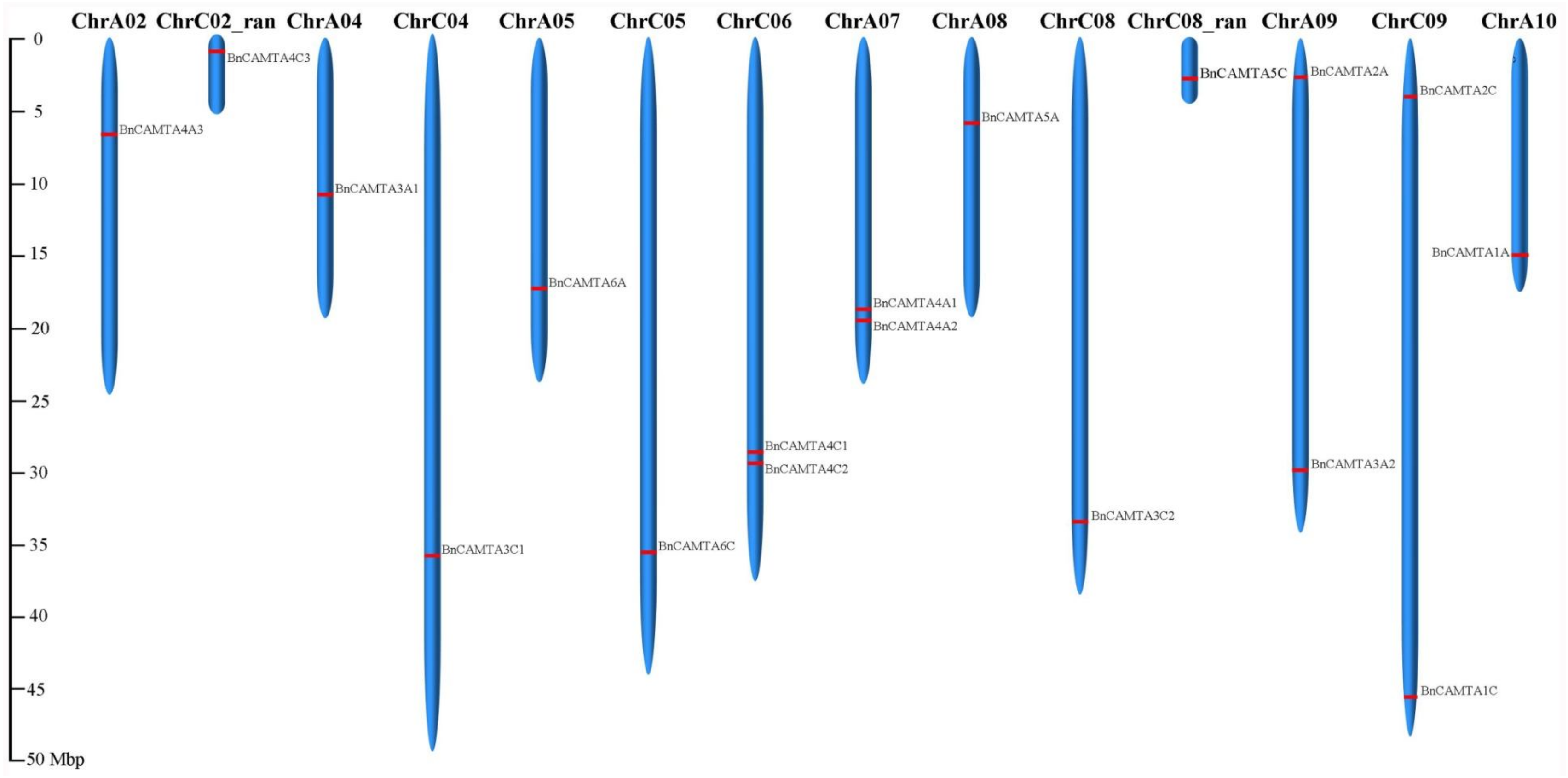

Figure S1 | Chromosomal distribution of 18 *CAMTA* genes in *Brassica napus*. The chromosome models are indicated in blue and drawn to scale. Chr02_ran and Chr08_ran represent ultra-contigs of chromosome 02 and chromosome 08, respectively that cannot be precisely mapped. Locations of the oilseed rape *CAMTA* genes in the chromosomes are shown as short absinthe-red lines.

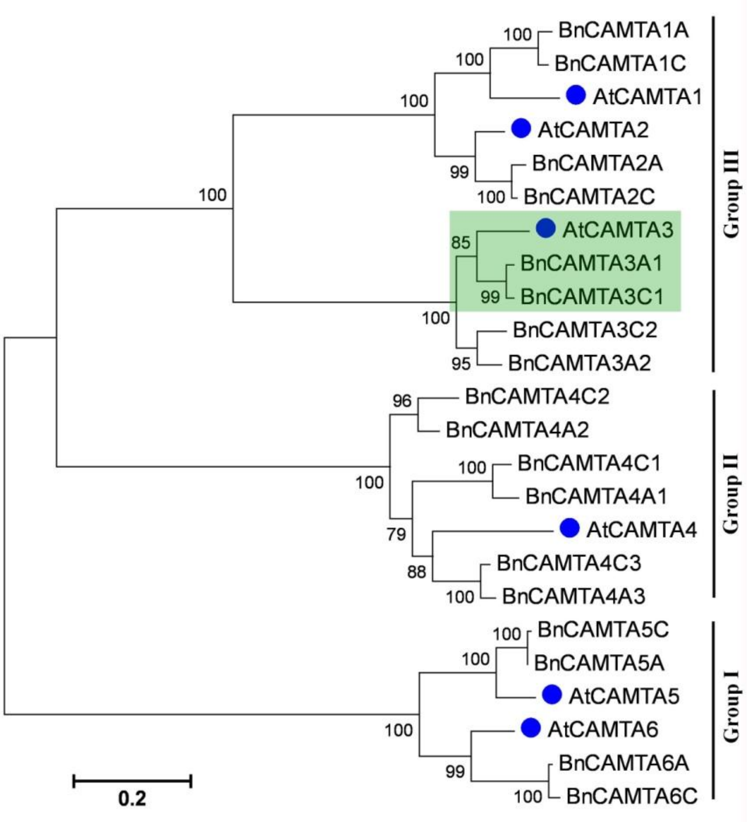

Figure S2 | Phylogenetic tree of CAMTAs from *B. napus* and Arabidopsis. Bootstrap values are displayed on the branches. Arabidopsis CAMTA proteins are marked with a solid blue circle before the protein names. The close relationship between AtCAMTA3 and BnCAMTA3s A1 and C1 reported in this study are highlighted in green box.

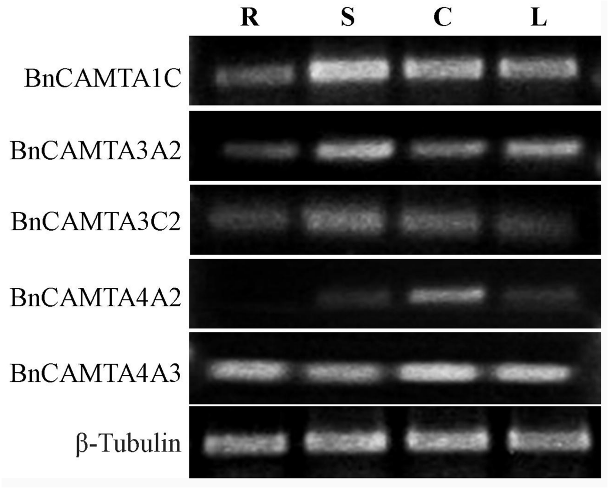

Figure S3 | Constitutive expression patterns of five *BnCAMTA* genes (1C, 3A2, 3C2, 4A2 and 4A3) in various tissues. Expression patterns of these genes in root (R), stem (S), cotyledon (C) and true leaf (L) were analyzed by second round of semiquantitative RT-PCR using first round RT-PCR product as template. The oilseed rape β-Tubulin gene served as a loading control gene. The profile of electrophoresis on a 1.5% agarose gel of the products obtained from 32 cycles (28 cycles for control) of PCR was shown.
